# Aqueous Extracts and Flavonoids Obtained from *Annona cherimola* Miller as Antidiabetic Treatments Alone and in Combination with Antidiabetic Drugs: In Vivo and In Silico Studies

**DOI:** 10.3390/ph18111754

**Published:** 2025-11-18

**Authors:** Jesica Ramírez-Santos, Fernando Calzada, Julita Martínez-Rodríguez, Miguel Valdes, Elizabeth Barbosa, Claudia Velázquez

**Affiliations:** 1 Unidad de Investigación Médica en Farmacología, UMAE Hospital de Especialidades, 2° Piso CORSE, Centro Médico Nacional Siglo XXI, Instituto Mexicano del Seguro Social, Av. Cuauhtémoc 330, Col. Doctores, Mexico City CP 06725, Mexico; jmr231289@gmail.com (J.M.-R.); valdesguevaramiguel@gmail.com (M.V.); 2 Instituto Politécnico Nacional, Sección de Estudios de Posgrado e Investigación, Escuela Superior de Medicina, Plan de San Luis y Salvador Díaz Mirón S/N, Col. Casco de Santo Tomás, Miguel Hidalgo, Mexico City CP 11340, Mexico; rebc78@yahoo.com.mx; 3 Laboratorio de Inmunología, Departamento de Sistemas Biológicos, Universidad Autónoma Metropolitana, Unidad Xochimilco, Calz. Del Hueso 1100, Col. Villa Quietud, Coyoacán CP 04960, Mexico; 4 Área Académica de Farmacia, Instituto de Ciencias de la Salud, Universidad Autónoma del Estado de Hidalgo, San Agustin Tlaxiaca CP 42076, Mexico; cvg09@yahoo.com

**Keywords:** *Annona cherimola*, antihyperglycemic, antihyperlipidemic, oral antidiabetic, flavonoids

## Abstract

**Background:** *Annona cherimola* Miller (*A. cherimola*) is traditionally used in Mexico to treat diabetes. **Objectives**: this study aimed to evaluate the antihyperglycemic activity of the aqueous leaf extracts (AEAcL) and stem (AEAcS) of *A. cherimola* alone and combined with oral antidiabetic drugs (OADs), as well as to determine their effect on % HbA1c, lipid parameters and toxicity. As well, the study aimed to isolate and identify some of its compounds to propose findings about its mode of action. **Methods**: Antihyperglycemic activity was evaluated using in vivo models with streptozotocin-induced experimental diabetes in Balb/c mice. Computer tools were used to obtain the pharmacokinetic and toxicological properties of the identified flavonoids; to obtain findings on their potential as α-glucosidase and SGLT1 inhibitors, in vivo and in silico studies were carried out using oral sucrose tolerance (OSTT) and glucose (OGTT) tests and molecular coupling studies. **Results**: ÇAEs and aSAAcS administered alone at 200 mg/kg showed a significant reduction in hyperglycemia. The best combination was AEAcL + Met (100/500 mg/kg), which significantly reduced hyperglycemic values and the % of HbA1c, TG, and LDL. The flavonoids isolated from AEAcL were identified as rutin, nicotiflorin, and narcissin. The molecular coupling assay and OSTT and OGTT tests showed that the flavonoids could inhibit α-glucosidase and SGLT1. **Conclusions**: AEAcL shows significant antihyperglycemic and antihyperlipidemic activity in murine models of diabetes, both alone (100 mg/kg) and in combination with metformin (100/500 mg/kg). Isolated flavonoids (rutin, nicotiflorin, and narcissine) appear to be partly responsible for these effects, although they have pharmacokinetic limitations. In silico and in vivo studies suggest a possible mechanism of action by inhibition of α-glucosidase and SGLT1.

## 1. Introduction

Diabetes mellitus (DM) is a chronic disease and a worldwide health problem. According to the World Health Organization (WHO), the number of people with this disease are alarming; in 1990, there were at least 200 million people, and in 2022, this number increased to 830 million [1]. In 2024, in Mexico, DM caused 57,986 deaths, according to data from the National Institute of Statistics and Geography [2]. Type 2 diabetes mellitus (T2DM) is a heterogeneous disease with variable clinical presentation and progression. More than 90% of patients with T2DM present with progressive loss of adequate insulin secretion by β cells. Moreover, the insulin resistance in tissues, and an inadequate compensatory insulin-secreting response, generates hyperglycemia in patients [3,4]. Long-term high blood glucose levels can result in advanced glycated end products (AGEs); their excessive production and accumulation in several tissues can cause damage to organs such as the heart, kidney, eyes, vasculature, and nerves [4,5,6,7,8,9]. One of the AGEs is glycated hemoglobin (HbA1c); this parameter is used as a screening test for the diagnosis of DM [10]. Additionally, it can be used as a witness of adequate control of glycemic values after a long-term pharmacological treatment. Another problematic aspect associated with DM is the inability of patients to synthesize fatty acids and triglycerides from carbohydrates or amino acids. This occurs due to the insufficient secretion or action of insulin, leading to the inhibition of the enzymes involved in these processes. Consequently, blood lipid disorders such as elevated levels of triglycerides, diacylglycerol, ceramides, free fatty acids, and low-density lipoprotein (LDL) cholesterol and a significative reduction of high-density lipoprotein (LDL) cholesterol are prevalent [11,12]. Type 2 diabetes mellitus involves a complex combination of metabolic, genetic, and environmental factors. Some of the modifiable risk factors that lead to the development of the disease are unhealthy diet, obesity, and sedentary lifestyle [13,14]; it has been reported that physical activity has three main benefits: the contraction of skeletal muscle cells that promote increased blood flow to the muscle, allowing the uptake of glucose from the plasma; the reduction of intra-abdominal fat; and the improvement of glucose uptake and insulin sensitivity [14]. Some non-modifiable factors are ethnicity and genetic predisposition; reports indicate that the Japanese, Hispanics, and Native Americans are the most at risk [4]. In addition, some research has provided convincing evidence of individual risk that T2DM is influenced by genetic factors, as whole genome studies have shown that T2DM has demonstrated the complex polygenic nature [15,16]. However, studies of association of the disease with the different loci are still needed.

The treatment of T2DM encompasses numerous oral medications, such as biguanides such as metformin, thiazolidinediones (TZDs) such as pioglitazone, rosiglitazone, and ciglitazone, sulfonylureas (SU) such as glibenclamide, dipeptidyl peptidase IV inhibitors (iDPP-IV), and sodium–glucose cotransporter 2 (SGLT2) inhibitors such as canaglifolzin [12]. However, several of the oral medications are known to produce side effects or hypersensitivity reactions in patients. Hypoglycemia, weight gain, gastrointestinal problems, and pancreatitis are some side effects of oral medicaments for T2DM [17]. In addition, in recent years, the prescription of TZDs has decreased due to unwanted effects such as fluid retention, edema, congestive heart failure, weight gain, skeletal fractures, and cancers [18]. Given the aforementioned factors, there has been a substantial increase in interest in developing novel treatments for T2DM, looking for effective agents for glycemic control, which delay the complications and avoid the probably side effects. In this sense, medicinal plants represent an alternative to treating some conditions [19]. The WHO has listed a total of 21,000 medicinal plants worldwide, including 400 available for the treatment of diabetes [20]. One of the families to which antihyperglycemic properties are attributed is the Annonaceae family; some of its species, such as *Annona diversifolia*, *Annona muricata*, *Annona aquamosa*, *Annona reticulata*, and *Annona cherimola*, have been studied and demonstrated potential antidiabetic activity [21]. *Annona cherimola* Miller (*A. cherimola*) is a small tree that produces fruits with economic importance [22]. It is distributed in Asia, South America, Europe, and Africa [21]. It is used in traditional Mexican medicine to treat several diseases, such as cough, fever, headache, inflammation, and diabetes [21,22,23]. Moreover, some authors have reported that its leaves possess antidepressant, pro-apoptotic, antihyperlipidemic, and antimicrobial properties [24,25,26,27]. Several compounds such as flavonoids, phenols, and alkaloids, among others, have been identified in *A. cherimola* leaves [22,28]. Previous studies of our research group demonstrate the antihyperglycemic and antilipidemic activity of ethanolic extract and aqueous extract [29,30,31]. The presence of the flavonoid rutin was identified in ethanolic extract, and the antihyperglycemic activity has been related to it. In addition, ethanolic extract has been evaluated in combination with antidiabetic drugs (OADs), being effective and helping to reduce significantly the hyperglycemic values [32,33]. However, the aqueous extract of *A. cherimola* leaves has not been evaluated in combination with OADs used in clinical practice. This is a crucial point in *A. cherimola* research, because several peoples traditionally consume this plant in infusions as an herbal remedy for DM2, but in some cases, they consume it in combination with OADs. Therefore, it is essential to analyze what happens when traditional Mexican medicine is combined with pharmacological treatment, to obtain more information about the use of this species and its efficacy as an antihyperglycemic agent.

Taking into account the above, the objective of this study was to evaluate the antihyperglycemic activity of aqueous leaf and stem extracts of *A. cherimola* alone and combined with AODs in acute models with type 2 diabetes induced by streptozotocin (ST2D); subsequently, subchronic studies were carried out on the aqueous extract with better acute activity, alone and in combination with OADs, to evaluate its antihyperglycemic capacity and its activity on some metabolic factor parameters (HbA1c and lipid profile). In addition, we isolated, identified, and evaluated the antihyperglycemic activity of some metabolites present in the aqueous extract and proposed a possible mechanism of action using silico strategies.

## 2. Results

### 2.1. Acute Oral Toxicity

The results obtained in the acute oral toxicity study indicated that the stem extract (AEAcS) of *A. cherimola* did not cause behavioral alterations or mortality in the animals during the observation period. At the end of the experiment, on day 14, gross evaluation of the internal organs showed no signs of tissue damage or significant weight loss. Therefore, AEAcS was classified within category 5 (LD_50_ > 3000 mg/kg), suggesting that its use is safe in humans.

### 2.2. Acute Evaluation of the Aqueous Extracts from Annona cherimola and Its Combinations

First, the acute effect of the aqueous extract of the leaves (AEAcL) and stem (AEAcS) from *Annona cherimola* was evaluated at 100, 200, and 300 mg/kg doses in ST2D mice with the aim of determining which was the most effective extract.

It was observed that the AEAcL, at doses of 100, 200, and 300 mg/kg, significantly reduces hyperglycemic levels from 1 to 7 h after treatment compared to the ST2D group, and the dose of 200 mg/kg was the most effective due to significantly decreasing the hyperglycemia compared to the initial values. With respect to the AEAcS, it shows significative difference when compared to ST2D; however, it does not reduce the hyperglycemic values compared to their initial values (Table 1).

Once the activity of the extracts was evaluated, the combination evaluations started with AEAcL. The first combination evaluated was with metformin; in this case, the best dose of AEAcL (200 mg/kg) was combined with the therapeutic dose of metformin (850 mg/kg). The combination of AEAcL + Met (200/850) reduced significantly the hyperglycemic values from 1 h to 7 h, reaching a hypoglycemic state. Regarding this, the decision to reduce the AEAcL dose to 100 mg/kg was made; in this case, the combination AEAcL + Met (100/850) showed a similar effect to the 200/850 combination, reaching hypoglycemic values. Thus, it was decided to reduce the metformin dose to 500 mg/kg and combine each one with 100 and 200 mg/kg of AEAcL. The combination of AEAcL + Met (200/500) continued generating hypoglycemia in the animals treated. On the other hand, AEAcL + Met (100/500) turned out to be more effective because it significantly reduced the hyperglycemia, reaching normoglycemic values without reaching hypoglycemia (Table 1).

Considering the results obtained in the metformin combination, the next combinations were evaluated with 100 mg/kg of AEAcL; in this case, the combination with acarbose (100/50) reduced significantly the hyperglycemic values from 0.5 to 5 h in comparison with the T2DM control and its initial values, indicating an effective combination. In the case of the combination of AEAcL + glibenclamide (100/5), this did not generate an effective reduction of hyperglycemia (Table 1).

To continue the investigation, the combination of AEAcS (100 mg/kg) with the OADs Met, Aca, and Gli. However, the effect observed after evaluation did not show a significant reduction in hyperglycemia; thus, it was decided to continue with the subchronic evaluations only with the combination of AEAcL and the OADs at the previously established doses.

### 2.3. Subchronic Evaluation of Aqueous Extract of A. cherimola Leaves and Its Combinations

Considering the acute results, in subchronical evaluation, it was decided to determine the activity of the best acute combination of AEAcL and metformin separately and as the combination AEAcL + Met, AEAcL + Aca, and AEAcL + Gli.

**Figure 1:**
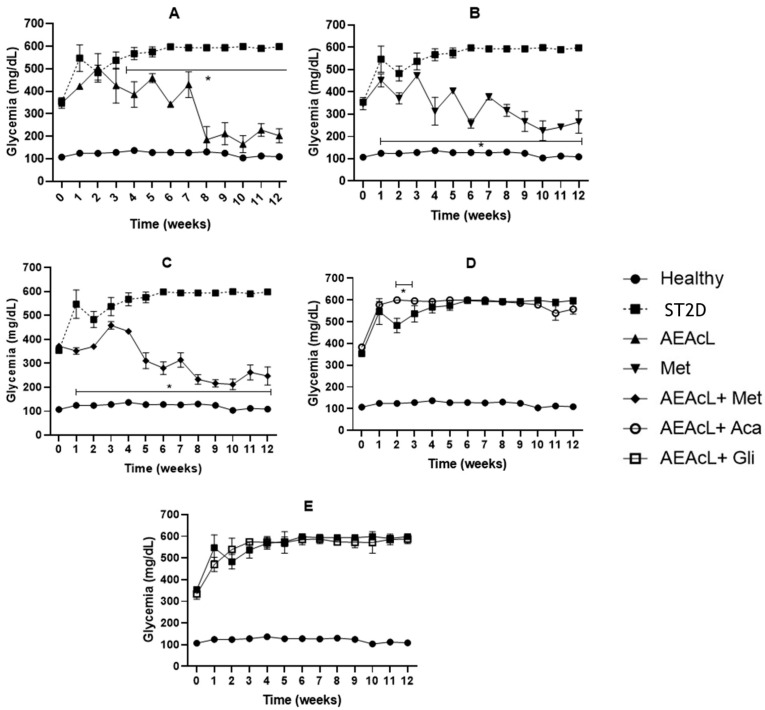
Results of subchronical evaluation of AEAcL 100 mg/kg (**A**), metformin 500 mg/kg (**B**), AEAcL + metformin 100/500 mg/kg (**C**), AEAcL + acarbose 100/50 mg/kg (**D**) and AEAcL + glibenclamide 100/5 mg/kg (**E**) in ST2D mice. Results are expressed as mean ± SEM, n = 6. * *p* < 0.05 vs. ST2D in the same week.

In respect to AEAcL, a significant decrease in hyperglycemia levels from weeks 3 to 12 was observed (Figure 1A). A similar activity was observed when metformin was evaluated separately; however, in this case, the significant reduction continued from weeks 1 to 12, reaching near to normoglycemic values from week 10 (Figure 1B).

When AEAcL + Met (100/500) was evaluated, a significant reduction was observed from weeks 1 to 12, reaching normoglycemic values from week 8 (Figure 1C). In respect to AEAcL + Aca and AEAcL + Gli (Figure 1D,E, respectively), these combinations did not generate an adequate control of hyperglycemic values, due to their activity loses from week 1 and they evolve like T2DM control.

### 2.4. Effects on % Glycated Hemoglobin After Subchronical Administration of Aqueous Extract of A. cherimola Leaves and Its Combinations

During the development of the subchronical assay previous shown, the % of glycated hemoglobin (%HbA1c) was determined at the beginning (0) and at weeks 4, 8, and 12. This measurement was done only on healthy, ST2D, AEAcL, metformin, and AEAcL + Met (100/500) groups, because they were the groups that showed a significant hyperglycemia reduction.

**Figure 2:**
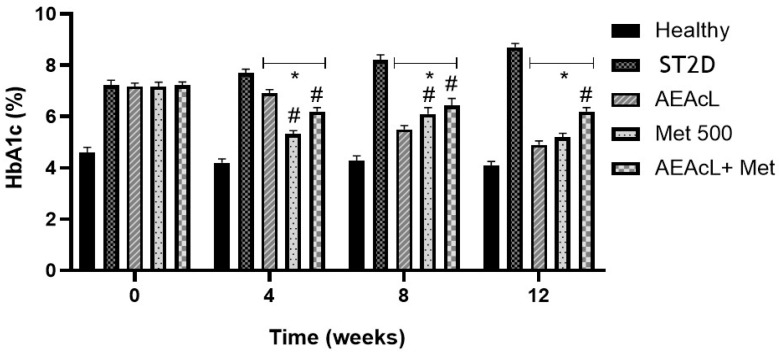
Percentage of glycated hemoglobin (% HbA1c) values at the beginning (0), 4, 8, and 12 weeks of subchronical assay. Results expressed as the mean ± SEM, n = 6; * *p* < 0.05 vs. ST2D; # *p* < 0.05 vs. healthy. ST2D: streptozocin-induced type 2 diabetic group; AEAcL: aqueous extract from *A. cherimola* leaves 100 mg/kg; Met: metformin 500 mg/kg; AEAcL + Met: combination of aqueous extract from *A. cherimola* leaves + metformin 100/500 mg/kg.

First, a significant increase in % HbA1c was observed in the ST2D group compared to healthy mice over the weeks; this represents the evolution of the T2D disease and the lack of hyperglycemic value control. On the other hand, a significant reduction of % HbA1c was observed in the AEAcL metformin and AEAcL + Met groups. This value was compared against the ST2D group during weeks 4, 8, and 12. Moreover, in week 4, it was observed that Met showed the best effect on the decrease in % HbA1c compared to AEAcL. In respect to AEAcL, this group showed the best effect in comparison with the other treatments in weeks 8 and 12. Finally, in week 12, the values obtained in the AEAcL group were near to those obtained from the healthy group; this is indicative of adequate hyperglycemic control (Figure 2).

### 2.5. Effect on Lipid Profile After Subchronical Administration of Aqueous Extract of A. cherimola Leaves and Its Combinations

Like %HbA1c, the lipid profile of the animals treated in subchronical assay was measured at the beginning (0), 4, 8, and 12 weeks. This profile was evaluated to observe the effect on cholesterol (CHO), triglycerides (TG), high-density lipoproteins (HDL), and low-density lipoproteins (LDL) after a subchronical administration of AEAcL, metformin, and their combination for a 12-week period.

The results for CHO showed that all groups obtained values like the healthy groups; only the group treated with metformin showed a significant increase compared to the healthy and ST2D groups on week 4 (Table 2). Regarding TG levels, ST2D showed a significant increase from weeks 4 to 12 compared to the healthy group. For its part, the AEAcL group showed an increased value along the assay; however, the values were shown to be significantly lower than ST2D. The Met group showed a significative increase compared to ST2D. On the other hand, the AEAcL + Met combination showed the best effect over this parameter, due to its significant reduction of the values compared to the ST2D group, reaching healthy values (Table 2). In respect to HDL-c and LDL-c, it was observed that the ST2D group decreased their HDL-c values and increased their LDL-c values significantly in respect to the healthy control. On the contrary, the treatment groups maintained values like the healthy control, with a significant increase in the Met group. Regarding LDL-c, the treatment groups maintained their values like the healthy control; however, the Met group significantly increased its values more than the ST2D control (Table 2).

Once the potential acute and subchronical antihyperglycemic activity of AEAcL was determined, the next step was to isolate and identify some of the metabolites present in the aqueous extract. The purification was performed by preparative thin-layer chromatography; three majority compounds were isolated and identified as follows.

### 2.6. Flavonoids Isolated from A. cherimola Leaves Aqueous Extract: Identification and Characterization

#### 2.6.1. HPLC-DAD Analysis of the Aqueous Extract of the Leaves of *Annona cherimola*

**Figure 3:**
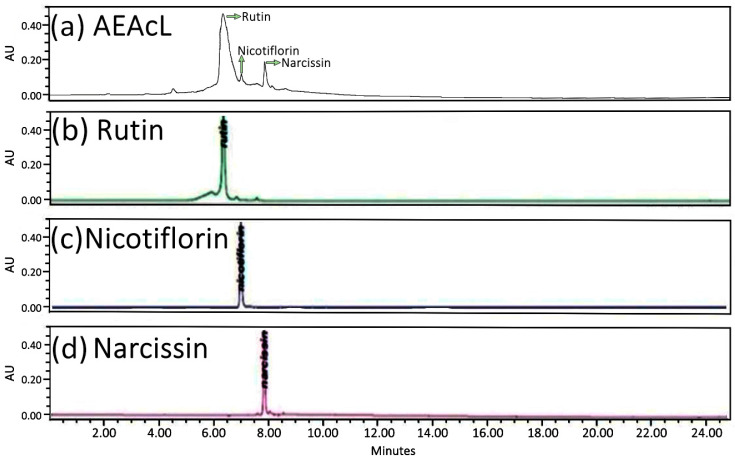
High-performance liquid chromatography analysis with diode array detection (HPLC-DAD) at 254 nm of (**a**) Aqueous extract of the leaves from *A. cherimola* (AEAcL) and the flavonoids standards (**b**) rutin, (**c**) nicotiflorin, and (**d**) narcissin.

First, the extract was subjected to analysis by high-performance liquid chromatography with diode array detection (HPLC-DAD). The analysis showed the presence of the flavonoids rutin, nicotiflorin, and narcissin; the flavonoid that obtained the highest abundance was rutin, with a retention time of 6.24 min, and those with the lowest abundance were nicotiflorin and narcissin, obtaining retention times of 7.19 and 7.75 min, respectively (Figure 3a). In addition, the presence of flavonoids was compared with the glycoside standards of the flavonoids rutin (Figure 3b), nicotiflorin (Figure 3c), and narcissin (Figure 3d).

#### 2.6.2. H and ^13^C-NMR Spectra Analysis of Rutin, Nicotiflorin, and Narcissin

Spectra of ^1^H-^13^C-NMR were obtained, and the identification of rutin, nicotiflorin, and narcissin was confirmed (Figures S1 and S2, Supplementary Materials).

**Rutin:** ^1^H-NMR (500 MHz, Chloroform-*d*) δ 6.37 (1H-6, d, *J* = 1.0), 6.21 (1H-8, d, *J* = 1.0), 7.62 (1H-2′, m, *J* = 0.92), 6.78 (1H-5′, d, *J* = 0.48), 7.50 (1H-6′, dd, *J* = 0.82), 5.04 (1H-1″, dt, *J* = 1.02), 3.63 (6H-2″, m, *J* = 7.33), 3.32 (H-3″, m, *J* = 1.67), 3.42 (1H-4″, m, *J* = 2.26), 2.48 (1H-5″, dt, *J* = 0.98), 3.63 (6H-6″, m, *J* = 7.33), 4.44 (1H-1′′′, m, *J* = 2.0), 3.63 (6H-2′′′, m, *J* = 7.33), 3.63 (6H-3′′′, m, *J* = 7.33), 3.32 (2H-4′′′, m, *J* = 1.67), 3.63 (6H-5′′′, m, *J* = 7.33), 0.98 (3H-6′′′, dd, *J* = 3.02). ^13^C-NMR (125 MHz, CDCl_3_) 157.32 (C-2), 134.17 (C-3), 178.26 (C-4), 161.84 (C-5), 99.25 (C-6), 65.02 (C-7), 94.34 (C-8), 157.47 (C-9), 104.79 (C-10), 122.11 (C-1′), 116.34 (C, C-2′), 145.56 (C-3′), 148.78 (C-4′), 116.06 (C-5′), 122.29 (C-6′), 102.58 (C-1″), 74.70 (C-2″), 76.82 (C-3″), 70.88 (C-4″), 76.65 (C-5″), 67.28 (C-6″), 101.60 (C-1′′′), 71.61 (C-2′′′), 71.77 (C-3′′′), 73.30 (C-4′′′), 69.35 (C-5′′′), 18.1 (C-6′′′).

**Nicotiflorin:** ^1^H-NMR (500 MHz, Chloroform-*d*) δ 6.21 (1H-6, d, *J* = 0.64), 6.37 (1H-8, d, *J* = 0.64), 8.12 (2H-2′, m, *J* = 1.25), 6.92 (2H-3′, m, *J* = 1.26), 6.92 (2H-5′, m, *J* = 1.26), 8.12 (2H-6′, m, *J* = 1.25), 5.34 (1H-1″, dt, *J* = 0.65), 3.63 (6H-2″, m, *J* = 4.71), 3.32 (2H-3″, m, *J* = 1.07), 3.92 (1H-4″, m, *J* = 1.43), 3.14 (1H-5″, dtt, *J* = 1.07), 3.63 (6H-6″, m, *J* = 4.71), 4.54 (1H-1′’’, m, *J* = 1.28), 3.63 (6H-2′′′, m, *J* = 4.71), 3.63 (6H-3′′′, m, *J* = 4.71), 3.32 (2H-4′′′, m, *J* = 1.07), 3.63 (6H-5′′′, m, *J* = 4.71), 1.26 (3H-6′′′, dd, *J* = 1.92). ^13^C-NMR (125 MHz, CDCl_3_). ^13^C NMR (125 MHz, CDCl_3_) 148.55 (C-2), 152.97 (C-3), 178.43 (C-4), 161.84 (C-5), 99.25 (C-6), 165.02 (C-7), 94.34 (C-8), 158.14 (C-9), 104.79 (C-10), 121.62 (C-1′) 133.97 (C-2′), 115.79 (C-3′), 160.15 (C-4′), 115.79 (C-5′), 136.40 (C-6′), 102.58 (C-1″), 74.70 (C-2″), 76.82 (C-3″), 70.88 (C-4″), 76.65 (C-5″), 57.28 (C-6″), 101.60 (C-1′′′), 71.61 (C-2′′′), 71.77 (C-3′′′), 73.30 (C-4′′′), 69.35 (C-5′′′), 18.10 (C-6′′′).

**Narcissin:** ^1^H-NMR (500 MHz, Chloroform-*d*) δ 6.22 (1H-6, d, *J* = 0.96), 6.87 (1H-8, d, *J* = 0.86), 8.16 (1H-2′, d, *J* = 0.84), OCH3 3.96 (3H, s), 6.15 (1H-5′, s), 7.84 (1H-6′, dd, *J* = 0.97), 5.34 (1H-1″, dt, *J* = 0.99), 3.55 (2H-2″, m, *J* = 2.04), 3.32 (2H-3″, m, *J* = 1.69), 3.92 (1H-4″, m, *J* = 2.22), 3.13 (1H-5″, dt, *J* = 0.85), 3.69 (2H-6″, dd, *J* = 1.96), 4.54 (1H-1′′′, m, *J* = 1,87), 3.76 (2H-2′′′, m, *J* = 1.96), 3.76 (2H-3′′′, m, *J* = 1.96), 3.32 (2H-4′′′, m, *J* = 1.69), 3.55 (2H-5′′′, m, *J* = 2.04), 1.26 (3H-6′′′, dd, *J* = 2.89). ^13^C-NMR (125 MHz, CDCl_3_) 157.38 (C-2), 134.13 (C-3), 178.26 (C-4), 161.84 (C-5), 99.25 (C-6), 165.02 (C-7), 94.34 (C-8), 157.47 (C-9), 104.79 (C-10), 122.12 (C-1′), 114.24 (C-2′), 148.60 (C-3′), OCH3 56.16, 149.01 (C-4′), 115.51 (C-5′), 122.77 (C-6′), 102.58 (C-1″), 74.70 (C-2″), 76.82 (C-3″), 70.88 (C-4″), 76.65 (C-5″), 67.28 (C-6″), 101.60 (C-1′′′), 71.61 (C-2′′′), 71.77 (C-3′′′), 73.30 (C-4′′′), 69.35 (C-5′′′), 18.10 (C-6′′′).

### 2.7. Acute Evaluation of Rutin, Nicotiflorin and Narcissin

To evaluate the acute effect of the flavonoids, glucose measurements at 2 and 4 h were considered, and metformin, acarbose, and glibenclamide were used as pharmacological controls to compare the activity of the flavonoids.

Acute evaluation of AEAcL showed a significant reduction in hyperglycemia compared to ST2D at 2 and 4 h (Table 3); similar activity was observed when OADs were evaluated. However, in the case of acarbose, it showed a significant reduction of hyperglycemic values in comparison to its initial values at 4 h. Moreover, glibenclamide showed a significant reduction of hyperglycemia at 2 h in comparison to its initial values. Finally, the flavonoids rutin, nicotiflorin, and narcissin had the best effect over inclusive hyperglycemic values—more effective than AEAcL. These reductions were significative at 2 and 4 h in comparison to the ST2D control and its initial values (Table 3).

Once the antihyperglycemic activity of the flavonoids was determined, the next step was to determine the probable action mechanism. In silico tools were used to determine the probable inhibitory activity of α-glucosidase and sodium–glucose cotransporter (SGLT-1).

### 2.8. Molecular Docking Studies of Rutin, Nicotiflorin, and Narcissin

**Figure 4:**
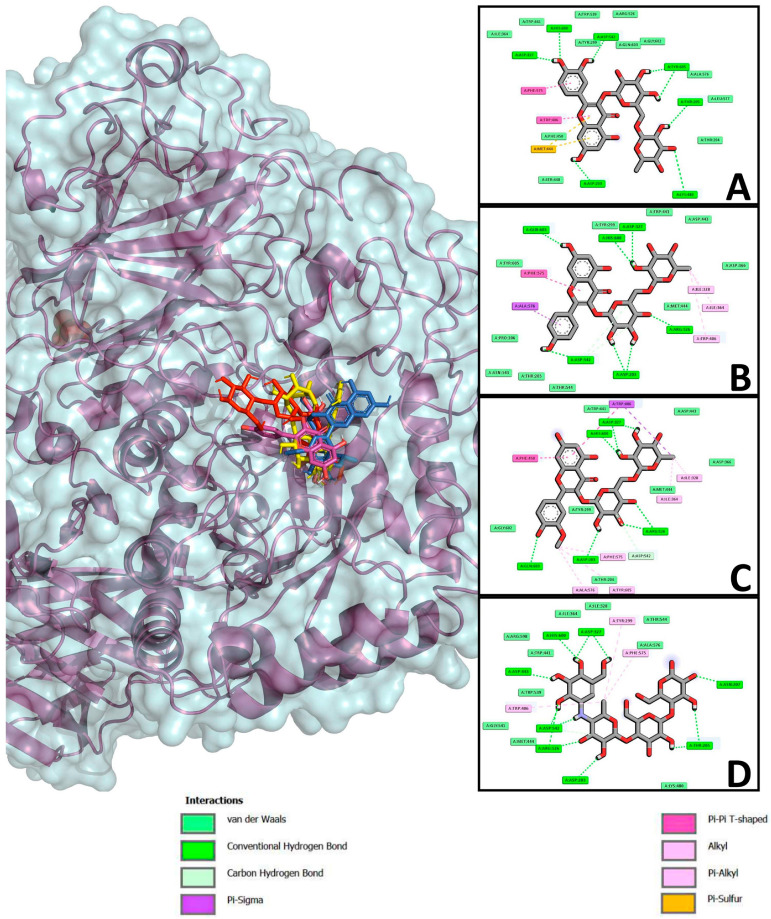
Results of molecular docking. The 2D images show the interactions obtained from the enzyme α-glucosidase and the binding of the flavonoids rutin (**A**), nicotiflorin (**B**), narcissin (**C**) and of acarbose (**D**); polar-type interactions are marked in green (conventional hydrogen bonds or carbon hydrogen bonds and van der Waals); non-polar interactions are marked in purple (pi-sigma), pink (alkyl or pi-alkyl), and orange (pi-sulfur). The 3D image shows the protein complex and the junction of rutin (yellow), nicotiflorin (magenta), narcissin (blue), and acarbose (red).

**Figure 5:**
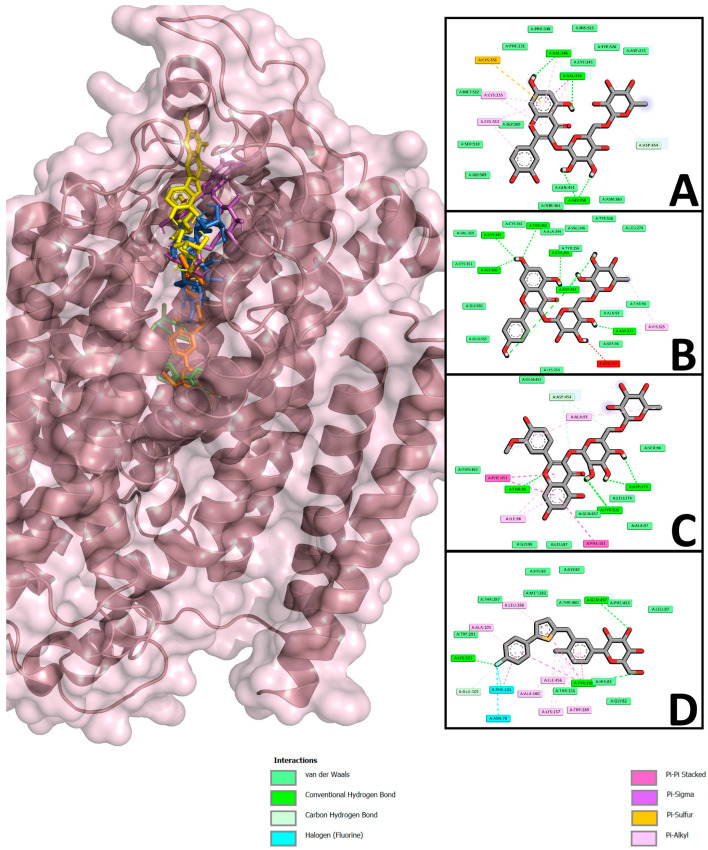
Results of molecular docking. The 2D images show the interactions obtained from SGLT1 and the binding of the flavonoids rutin (**A**), nicotiflorin (**B**), narcissin (**C**) and of canagliflozin (**D**); polar-type interactions are marked in green (conventional hydrogen bonds or carbon hydrogen bonds and van der Waals); And non-polar interactions are marked in purple (pi-sigma), pink (alkyl or pi-alkyl), and orange (pi-sulfur). The 3D image shows the protein complex and the junction of rutin (yellow), nicotiflorin (magenta), narcissin (blue), and canagliflozin (orange) are represented.

Molecular coupling was performed to determine the possible interaction of the flavonoids with the enzyme α-glucosidase and SGLT1 (Table 4, Figure 4 and Figure 5), two important targets in the therapeutic management of DM. The enzyme α-glucosidase participates in the hydrolysis of complex carbohydrates [34] and SGLT1 (sodium cotransporter glucose 1) is described as responsible for glucose uptake in the body [35]. The results of the interaction of the flavonoids and α-glucosidase showed that, overall, all three flavonoids obtained a better affinity for the enzyme compared to the reference drug acarbose. The flavonoids narcissin, nicotiflorin, and rutin obtained binding energies of −5.61, −5.23 and −4.44 kcal/mol, respectively; these values were compared to that obtained with the reference drug acarbose, which was −4.36 kcal/mol (Table 4, Figure 4). The interactions obtained showed that the flavonoids shared several amino acids of the enzyme’s active site and also shared polar and nonpolar interactions with acarbose; narcissin and nicotiflorin shared 10 polar interactions (Asp 203, Thr 205 or Thr 204, Tyr 299, Asp 327, Asp 443, Met 444, Arg 526, Asp 542, Thr 544, and His 600, respectively) and 5 nonpolar interactions (Ile 328, Ile 364, Trp 406, Phe 575, and Ala 576); the routine shared 12 polar interactions (Asp 203, Thr 204, Thr 205, Tyr 299, Asp 327, Ile 364, Trp 441, Lys 480, Arg 526, Trp 539, Asp 542, Ala 576, and His 600) and 3 nonpolar interactions (Trp 406, Met 444, and Phe 575).

For the SGLT1 results, the flavonoids did not obtain favorable binding energies compared to the control drug canagliflozin, which obtained a binding energy of −6.77 kcal/mol. Rutin, nicotiflorin, and narcissin obtained binding energies of 24.12, −2.15, and −1.17, respectively (Table 4, Figure 5).

### 2.9. Oral Sucrose Tolerance Test (OSTT)

**Figure 6:**
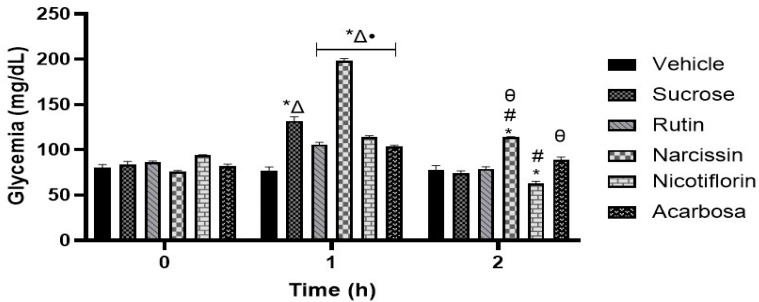
Effect of flavonoids on the oral sucrose tolerance test (OSTT). Data are expressed as means ± SEM, n = 6. * *p* < 0.05 vs. initial values; ^Δ^ *p* < 0.05 vs. vehicle for 1 h; # *p* < 0.05 vs. vehicle for 2 h; • *p* < 0.05 vs. sucrose for 1 h; θ *p* < 0.05 vs. sucrose for 2 h. Groups treated with vehicle, sucrose (3 g/kg), rutin, nicotiflorin, narcissin, and acarbose (50 mg/kg).

The administration of rutin, nicotiflorin, and acarbose in the OSTT assay showed a reduction in the postprandial peak at the first hour compared to the sucrose group (Figure 6). The results obtained suggest that the antihyperglycemic activity of rutin and nicotiflorin is mediated by the inhibition of the hydrolysis of complex carbohydrates with glycosidic bond types α-1,4 and β-1,4. Meanwhile, narcissin produced an increase in the postprandial peak at the first hour and an increase in glucose levels at two hours compared to the group treated with the vehicle.

### 2.10. Oral Glucose Tolerance Test (OGTT)

**Figure 7:**
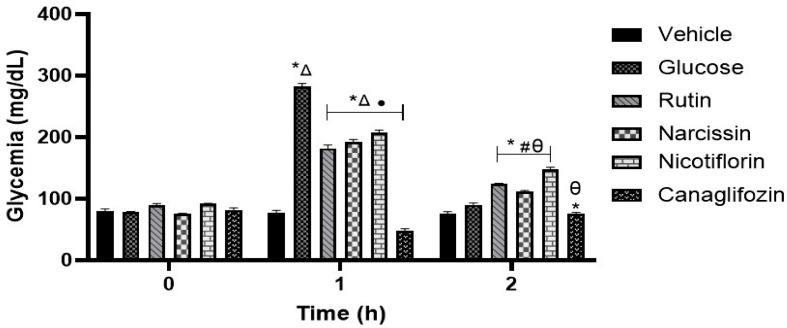
Effect of flavonoids on the oral glucose tolerance (OGTT) test. Data are expressed as means ± SEM, n = 6. * *p* < 0.05 vs. initial values; ^Δ^ *p* < 0.05 vs. vehicle for 1 h; # *p* < 0.05 vs. vehicle for 2 h; • *p* < 0.05 vs. glucose for 1 h; θ *p* < 0.05 vs. glucose for 2 h. Groups treated with vehicle, glucose (3 g/kg), rutin, nicotiflorin, narcissin, and canagliflozin (50 mg/kg).

After administration of rutin, narcissin, and nicotiflorin on OGTT, a reduction in the postprandial peak was obtained in the first hour; however, the greatest reduction in the postprandial peak was obtained with canagliflozin. Assessments at 2 h showed an increase in glucose levels in the groups treated with rutin, narcissin, and nicotiflorin compared to the vehicle-treated group (Figure 7). The results obtained suggest a possible inhibition of the three flavonoids on SGLT1.

### 2.11. Toxicoinformatic and Pharmaceutical Analysis of Flavonoids

A computational evaluation of the physicochemical, pharmacokinetic, and toxicological properties of rutin, nicotiflorin, and narcissin was performed (Table 5). The results are summarized in Table 5. Regarding physicochemical characteristics, the three flavonoids presented a high topological surface polar area (TPSA) (rutin: 269.43 Å^2^; nicotiflorin: 249.2 Å^2^; narcissin: 258.4 Å^2^), a considerable number of rotatable bonds (6–7), as well as multiple donors (9–10) and acceptors (15–16) of hydrogen bonds. Lipophilicity values (logP) were low, ranging from 0.72 to 1.16, indicating a low affinity for lipid membranes. The estimated water solubility (logS) was moderately low in all cases. In addition, all the compounds violated multiple selection rules for molecules with oral pharmacological potential. In Lipinski’s rule, the three flavonoids presented three violations (molecular weight > 500 Da, number of donors > 5, and acceptors > 10). Likewise, according to the criteria of Ghose, Veber, and Egan, infractions associated with molecular size, polarity, and number of atoms were also observed. In pharmacokinetic analysis, rutin showed medium intestinal absorption, while nicotiflorin and narcissin exceeded this threshold. None of the compounds demonstrated potential to cross the blood–brain barrier. The estimated volume of distribution was similar among the three flavonoids, and the percentage of plasma protein binding was high (rutin: 85.0%; nicotiflorin: 85.1%; narcissin: 84.5%). None of the compounds were identified as inhibitors or substrates of the main cytochrome P450 isoforms. Clearance was classified as low, and the plasma half-life (T1/2) was estimated to be between 4.27 and 4.6 h. The in silico evaluation of the toxicity of the flavonoids rutin, nicotiflorin, and narcissin revealed a favorable safety profile for all three compounds. According to the predictive models used, none of the flavonoids showed mutagenic, carcinogenic, or neurotoxic potential. In addition, the toxicological classification of all terpenoids indicated that they belonged to class V, indicating they can be harmful if ingested.

## 3. Discussion

Diabetes mellitus is a heterogeneous chronic disease with a high prevalence rate worldwide [36]. It is characterized by hyperglycemia resulting from defects in insulin secretion, insulin action, or both [37]. In the long term, hyperglycemia leads to damage and dysfunction of various organs such as the eyes, kidneys, heart, nerves, and blood vessels [36,37]. There are several treatments for DM; however, current medications have limitations for the therapeutic management of the disease, and their prolonged use leads to adverse effects [38]. One of the metabolic alterations that accompanies DM is dyslipidemia, the most frequent hyperlipidemia that increases the risk of atherosclerotic vascular disease [39]. It has been reported that metabolic disorders in DM could imply an increase in triglyceride (TG) and low-density lipoprotein (LDL-C) levels, which could lead to acute myocardial infarction and cardiovascular risks [39,40]. Therefore, the search for alternatives for the treatment of DM remains a challenge in research.

One of the most relevant sources for obtaining molecules with anti-hyperglycemic activity are medicinal plants. Numerous studies have reported on the effects of a large number of plant species to combat DM and that the molecules to which this pharmacological effect is attributed are bioactive compounds such as flavonoids, carotenoids, terpenoids, alkaloids, and glycosides [23,41].

The aim of this study was to determine the antihyperglycemic effect of two aqueous extracts of the leaves and stem of *A. cherimola*, as a treatment alone and in combination with oral antidiabetic drugs (OADs). Subsequently, the extract with the best acute activity was subjected to a subchronic study to obtain information on its effect on glycemic values, HbA1c, and lipid profile. In addition, based on the aqueous extract of *A. cherimola* leaves (AEAcL), three flavonoids were identified, and their acute antihyperglycemic activity was evaluated, and, finally, a molecular docking was performed to determine their possible interaction with the enzyme α-glucosidase and the SGLT1 cotransporter. It is important to note that all experiments were conducted in female mice because, in Mexico, a higher prevalence of DM2 has been observed in females than in males—20.1 and 16.3%, respectively [42]. In addition, previous studies have evaluated the antihyperglycemic activity of *A. cherimolla* in male mice only and in both sexes [30,31,32,33].

First, the acute oral toxicity of *A. cherimola* aqueous stem extract (AEAcS) was evaluated, classifying it as category 5 according to the criteria established by the OECD. This classification indicates a low level of toxicity and suggests that the extract is potentially safe for use in humans. Likewise, this category implies a wide range of safe doses for administration in animal models. During the observation period, no clinical signs of toxicity were detected, and gross necropsy revealed no obvious pathological alterations in the internal organs. On the other hand, acute oral toxicity data for *A. cherimola* leaf extract were published in a previous paper and indicated that an LD_50_> 3000 mg/kg was obtained [30].

Subsequently, we evaluated the acute effect of aqueous extract of *A. cherimola* leaves and stem (AEAcL and AEAcS, respectively) (Table 1). Considering previous studies to obtain an antihyperglycemic effect from ethanolic and aqueous extracts, doses at 100, 200, and 300 mg/kg were considered [29,30,31,32,33]. The results showed that the best effect on the reduction of hyperglycemia was obtained from AEAcL at the dose of 200 mg/kg, since a slight decrease in baseline glucose values was observed against all measurements of the acute assessment and a significant decrease was observed compared to the values of the ST2D group. In addition, when comparing them with the hyperglycemic values of the ST2D group, it was observed that both extracts obtained significant effects on lowering glycemia for 3.5 and 7 h after the administration of the treatment at doses of 100, 200, and 300 mg/kg. Previous studies support the effect obtained by both *A. cherimola extracts* evaluated, as the hyperglycemia-reducing effect of ethanolic and aqueous extracts has been demonstrated in animals with type 2 diabetes induced by streptozotocin or alloxane [29,30,31,32,33,42]. Regarding the combinations of the extracts with oral antidiabetic drugs, the dosage of each drug and previous studies where experimental animals were used were considered for the doses used in the experiments [29,30,31,32,33,43,44,45]. First, treatments with the combinations of AEAcL + metformin were performed. The doses of 100/850, 200/850, and 300/850 mg/kg were used; however, the animals presented hypoglycemia during the last measurements of the acute study (Table 1). These results led us to decrease the dose of metformin; the combination of AEAcL + metformin at a dose of 100/500 mg/kg had the best effect by significantly reducing hyperglycemia from 1 to 7 h of the study, compared to ST2D. The results for the combinations of AEAcL+ Aca (100/50 mg/kg) and AEAcL+ Gli (100/5 mg/kg) obtained significant decreases against ST2D at 1, 3, and 5 h. For AEAcS + Met (100/500 mg/kg), the results showed that hyperglycemic values decreased significantly compared to the ST2D group throughout the study; however, hypoglycemia developed at 3 h after treatment administration. Combinations of AEAcS + Aca (100/50 mg/kg) also significantly reduced hyperglycemia values compared to ST2D from 0.5 to 7 h after administration. It could be inferred that the reducing effect of hyperglycemia in all cases is due to a synergism of AEAcL and AEAcS in combination with OADs. One study supports observation, as ethanolic extract of *A. cherimola* leaves has been shown to have effective results on hyperglycemia as a treatment alone and in combination with OADs in an acute model [32].

Considering the results, we decided to select the combination of AEAcL + Met (100/500 mg/kg) to perform the chronic study (Figure 1). Individual assessment of the AEAcL and metformin resulted in a significant reduction in hyperglycemia. However, the combination of AEAcL + Met (100/500 mg/kg) showed a better effect by reducing hyperglycemia from week 1, and against its baseline values from week 5, until the end of the study. The data obtained suggest that the presence of metabolites in the AEAcL favors a synergistic activity with Met, since a better reduction of hyperglycemia is achieved in less time compared to their individual evaluation. As for the combinations of AEAcL + Aca (100/50 mg/kg) and AEAcL + Gli (100/5 mg/kg), these did not generate adequate control of hyperglycemia. One report supports our results, as the loss of effect of glibenclamide in combination with ethanolic extract of *A. cherimola* leaves at 300 mg/kg was reported in an 8-week subchronic study. In addition, for Acarbose, it was indicated that in week 4, it caused the death of animals [32]. The loss of effect is due to the phenomena of pharmacological tolerance, saturation of the mechanisms involved, functional depletion of β pancreatic cells, or pharmacokinetic interactions between the extract and conventional drugs. Studies have shown that antidiabetic herb–drug interactions can improve or compromise glycemic control; therefore, our findings of loss of efficacy in some combinations are consistent with the literature that warns of both synergies and risks of pharmacokinetic/pharmacodynamic interaction [46].

Also, the percentage of glycated hemoglobin (% HbA1c) was measured for each treatment group (Figure 2). For this purpose, only the evaluation of AEAcL (100 mg/kg), Met (500 mg/kg), and AEAcL + Met (100/500 mg/kg) was considered as having good antihyperglycemic effect in the chronic study. The results showed that the three treatments mentioned obtained a significant reduction in the values of % HbA1c compared to ST2D. The data obtained were consistent with the reduction of hyperglycemia, as it has been reported that the reduction of glucose in bloodstream results in a decrease in the formation of Shiff bases, amadori products, and subsequent oxidative modifications to produce advanced glycosylation products (EFAs) [47].

On the other hand, during the study, lipid profile values were measured (Table 2), and the groups treated with 100 mg/kg and 100/500 mg/kg (100/500 mg/kg) resulted in good effects by maintaining values close to the healthy group for the parameters of CHO, HDL-c, and LDL-c throughout the study; TG values decreased significantly compared to the ST2D group, while the ST2D group obtained a significant increase in TG and LDL-c values and a significant decrease in HDL-c compared to healthy animals. Some studies support our results, as it has been reported that the leaves of *A. cherimola* have activity against hyperlipidemia, decreasing the concentration of CHO, TG, and LDL-C [32,34,36]; in addition, it has been reported that from a decoction of the leaves, a reduction in cholesterol absorption was obtained by decreasing the activity of the enzyme HMG-CoA reductase in in vitro experiments [48].

Subsequently, the purification of the AEAcL was performed by preparative thin-layer chromatography, and three flavonoids were identified by high-performance liquid chromatography with diode array detection (HPLC-DAD) (Figure 3) and spectroscopic methods of 1H and 13C NMR (Figures S1 and S2, Supplementary Materials). The results confirmed the presence and identification of rutin, nicotiflorin, and narcissin in the aSAAcL. The results are supported by other reports where the presence of these flavonoids was identified in the leaf extract of *A. cherimola* [30]. In addition, the identification of rutin and other phenolic compounds in higher abundance in ethanolic extracts of *A. cherimola* has been reported [22]. And a seasonal analysis of this species has been carried out where, in the months of May, June, July, and August, it was observed that the flavonoid rutin is in the majority in all the months of collection [31]. Therefore, in our study, the extract collected in the month of December was used, since in previous studies, extracts from this month’s collection were used [29,32,33]. On the other hand, it is important to consider that more complete seasonal and geographical studies would be needed, since differences in the concentration of metabolites present in the extract could cause modifications in the biological activity of the plant material.

The next step of our study was the evaluation of the antihyperglycemic effect of the flavonoids identified in the AEAcL. The data obtained showed that the flavonoids obtained a better effect on the reduction of hyperglycemia compared to OADs. The rutin, nicotinflorin, and narcissin antihyperglycemic activity is supported in another study [35]. Moreover, the activity of flavonoids isolated from plant species has been shown to have beneficial effects on diabetes by improving blood glucose level, lipid profiles, and antioxidant status [49,50]. One of the flavonoids that has been identified in numerous plant species with antidiabetic properties is rutin [51,52], as its mechanisms of action have been proposed to include decreasing the absorption of carbohydrates from the small intestine, increasing tissue glucose uptake, inhibition of tissue gluconeogenesis, and stimulating insulin secretion from beta cells. It may also be a protector of Langerhans islets [50,51,52].

Once the potential antidiabetic activity of the three isolated flavonoids was demonstrated, in silico studies were carried out to propose two probable action mechanisms; moreover, in vivo oral glucose and sucrose tolerance tests were carried out to corroborate the in silico findings (Table 4, Figure 4, Figure 5, Figure 6 and Figure 7). First, in silico results on α-glucosidase enzyme showed that the three flavonoids had a binding pocket and position like the control drug acarbose. Moreover, narcissin, nicotiflorin, and rutin shared a similar binding site with two relevant polar interactions with Asp 443 and Met 444 amino acid residues. These in silico findings provide a preliminary insight into the probable antidiabetic mechanism of action mediated by the three flavonoids; however, in vivo studies were carried out to reinforce our hypothesis. In this case, the oral sucrose tolerance test showed that rutin and nicotiflorin generate an attenuation of the postprandial peak, evidencing a possible inhibitory effect on the digestion or absorption of sucrose or an improvement in peripheral glucose uptake. At hour 2, glucose levels in the nicotiflorin-treated group were significantly lower compared to the sucrose group; rutin reached values close to the group treated with acarbose, a reference drug with inhibitory action on intestinal α-glucosidases. These results suggest that the flavonoids evaluated could act by a comparable mechanism, delaying the release of glucose into the systemic circulation. Other studies have also reported a favorable interaction of rutin and nicotiflorin on the enzyme α-glucosidase [32,53,54]. The inhibition of this enzyme using rutin as a drug has been demonstrated by oral sucrose tolerance assays in vivo; therefore, it supports what was found in our work [33]. In addition, there are several in vitro studies using enzyme assays that support our results. All of them show that rutin, narcissin, and nicotiflorin inhibit the α-glucosidase enzyme with competitive mechanisms and significant affinity like acarbose [54,55,56,57,58,59]. Our investigation reinforces this hypothesis and supports the in vitro information described for rutin, narcissin, and nicotiflorin as antidiabetic agents.

The second mechanism of action proposed was as an SGLT1 inhibitor. In silico analysis showed that canagliflozin obtained the best binding energy, and its affinity was favorable. While the flavonoids did not obtain favorable binding energies, this could be due to a steric impediment from the presence of diglycosides in the chemical structures of rutin, nicotiflorin, and narcissin. Similar results have been reported showing unfavorable binding energy using rutin as ligand for SGLT1 [32]. To determine the probable SGLT inhibition, in vivo oral glucose tolerance tests were carried out. The results corroborate those obtained from in silico assays, since the three flavonoids significantly reduced the postprandial peak at the first hour compared to the group treated with glucose; however, the results obtained showed a significant increase of glucose compared to the initial values and vehicle group. Moreover, the inhibition was lower compared to those obtained in the canagliflozin group. More studies are needed to confirm these findings and explore the exact mechanisms by which flavonoids might be exerting this effect.

The in silico analysis of the physicochemical, pharmacokinetic, and pharmacological properties of the flavonoids rutin, nicotiflorin, and narcissin allowed us to identify predictions about some limitations and potential advantages in their use as bioactive candidates (Table 5) [60,61,62,63]. Structurally, all three compounds share a high degree of polarity (TPSA > 249 Å^2^) and a considerable number of hydrogen bond donors and acceptors, as well as molecular weights of more than 500 Da. These characteristics contribute to their low passive permeability across biological membranes, including the blood–brain barrier (BBB), which was corroborated by the predictive models used. In addition, all the flavonoids violated multiple classic drug similarity rules, including those of Lipinski, Ghose, Veber, and Egan, suggesting low oral bioavailability if administered without structural or technological modifications. Regarding pharmacokinetic properties, rutin showed a mean intestinal absorption, while nicotiflorin and narcissin had optimal values, indicating a better relative absorption according to the prediction of bioinformatics tools. However, none of the three compounds showed the ability to cross the BBB, which limits their therapeutic applicability in pathologies of the central nervous system, although it may be an advantage in treatments where adverse effects at the brain level are to be avoided. On the other hand, all three compounds have a high affinity for plasma proteins (~85%), which can reduce the free fraction available in the systemic circulation. However, none of the flavonoids evaluated showed potential for inhibition or interaction with the main cytochrome P450 isoforms (CYP1A2, CYP2C9, CYP2C19, CYP2D6, and CYP3A4), which represents an advantage from the point of view of metabolic safety and low risk of drug interactions. The estimated half-life of these compounds ranges from 4.27 to 4.6 h, indicating that sustained-release formulations or multiple daily doses may be necessary to maintain effective plasma concentrations. Some in vivo and in vitro studies support the low oral bioavailability of rutin [62,63] and nicotiflorin due to their low intestinal permeability and metabolism in the intestinal flora [64]. However, complete pharmacokinetic studies would be needed to confirm the pharmacokinetic results obtained using bioinformatics tools.

Regarding the toxicology of the flavonoids, the computer tools showed a low risk of adverse effects related to genetic alterations, tumor development, or toxicity at the level of the central nervous system and a classification of 5, indicating that they can be harmful if ingested. This correlated with what was obtained in the acute oral toxicology test. However, subchronic toxicity testing is necessary to confirm the low toxicity of AEAcL. It should be noted that, at the end of each test, a gross necropsy was performed to verify if the stomach, intestine, spleen, liver, and kidney organs presented any variability in terms of weight, coloration, or tissue damage; however, there was no variation compared to the control group. On the other hand, some data obtained in a subchronic study to determine the antihyperglycemic activity support the findings, as it was demonstrated by histology that the aqueous extract of the leaves of *A. cherimola* did not generate alterations in the liver and kidney and that it generated significant improvements in the alterations associated with diabetes [31].

The results obtained demonstrate the importance of flavonoids as antihyperglycemic agents. Phytochemical studies show that both flavonoids and their derivatives could have a therapeutic impact on the treatment of T2DM. An example are the isocoumarins, which can act as α-glucosidase inhibitors, and those structural modifications in the aromatic or lactonic core (hydroxylation, methoxylation, glycosylation) strongly modulate the potency and mode of inhibition [65]. Recent work synthesized six flavonoid derivatives modified with phenylpropionic acid (esterification) and demonstrated potent inhibitory activity on α-glucosidase [66].

Moreover, flavonoids represent an important source of effective antidiabetic treatments that have been reported over the years by several researchers [67,68]. Some of them, such as resveratrol, rutin, catechin, and epicatechin, are leader molecules used in the present day in translational research looking for new treatments for DM2 based on natural sources [69,70].

The complete analysis of the evaluations performed in this work provides information on the antihyperglycemic and antihyperlipidemic activities of AEAcL as treatment, alone and in combination with OADs, as well as the antihyperglycemic activity of its identified flavonoids. In addition, it provides information on the possible mechanism of action by which the antihyperglycemic activity of flavonoids could be mediated. However, more studies are needed to know the effect of combining the use of *A. cherimola* with other OADs and flavonoids with OADs.

## 4. Materials and Methods

### 4.1. Reagents, Drugs, and Chemicals

Streptozocin (≥75% α-anomer basis, PN: S0130-5G), metformin (PN: PHR1084-500MG), nicotinamide (≥99.5%, PN: 47865-U), acarbose (PN: PHR1253-500MG), glibenclamide (PN: PHR1287-1G), acetonitrile, ethanol, and acetic acid of HPLC grade were purchased from Sigma-Aldrich^®^ (Sigma^®^, Saint Louis, MO, USA). TLC glass plates, L × W 20 cm × 20 cm, and sílica gel 60 F254, 2 mm (CC: Z292974), were purchased from Merck^®^ (Merck^®^, Darmstadt, Germany). Ethanol anhydrous (CC: 15568604), buffer solution (citric acid/sodium hydroxide/hydrogen chloride, pH 4.00, CC: 109445), dichloromethane (CC:15594055), methanol (CC:10284580), ethyl acetate (CC:10382681), and solvents were purchased from J.T. BakerTM (Thermo Fisher Scientific, Waltham, MA, USA).

### 4.2. Plant Material

The plant material was collected by Dr. Fernando Calzada in December 2023 in San José, Tláhuac, Mexico (19°16′32.6” N 99°00′07.1” W). The plant material was authenticated by Santiago Xolalpa, from the IMSSM Herbarium of the Mexican Institute of Social Security (IMSS); the voucher identification is 15795.

### 4.3. Obtention of Aqueous Extracts of the Leaves and Stems from Annona cherimola

Infusions were made for the preparation of extracts of the leaves and stem from *A. cherimola*; in both cases, 1.5 g of herb material powder was placed inside individual envelopes of food-grade filter paper, which were sealed and placed for 20 min inside 125 mL of previously boiled purified water, to finally be removed and discarded, preserving the extract in the infusion obtained. Extracts were then filtered and evaporated to dryness in a rota-evaporator (Büchi Labortechnik AG, Flawil, Switzerland) at a reduced pressure of 40 °C. Once dried and at a constant weight, the extracts were stored refrigerated at 4 °C in sealed bottles until the time of use. The yield obtained for the leaf extract was 357.58 mg, and for the stem, it was 295.70 mg/kg.

### 4.4. Isolation, Extraction, and Identification of Flavonoids

The isolation and purification of the flavonoids was performed by preparative thin-layer chromatography (Merck 60F-254 silica gel). A portion (300 mg) was purified using a mixture of EtOAc:MeOH:water (10:1,6:1,3) to obtain narcissin (40 mg), nicotiflorin (38.4 mg), and rutin (75.5 mg). The process was repeated as needed.

For the identification and characterization of the flavonoids, an HPLC analysis with diode array (DAD) detection (Waters Agilent, 5301 Stevens Creek Blvd, Santa Clara, CA, USA) was performed, equipped with a 250 mm × 4.6 mm C18 analytical column (Waters) and a particle size of 5 μm (Waters) (Spherisorb S50D52, Waters Corporation, Milford, MA, USA). For this, 10 mg of the aqueous extract of the leaves of *Annona cherimola* (AEAcL) was used and dissolved in 10 mL of ethanol; for the analysis, 20 μL was injected. The system used was composed of a binary mobile phase composed of the solvent 2% acetonitrile/acetic acid in water (A) and 100% acetonitrile (B). Gradients were used in 4 stages; first stage: linear gradient of 80 (A)/20 (B) for 8 min; second stage: linear gradient of 40/60 for 5 min; third stage: linear gradient of 30/70 for 6 min; fourth stage: 90/10 linear gradient for 6 min, with a flow rate of 1 mL/min mobile phase. The detection was made at a wavelength (λ) of 254 nm at room temperature, and the total elution time was 25 min. At the end, the data obtained were graphed. The retention times obtained from the AEAcL and UV spectra were compared with the standards (Figure 3).

Also, products obtained from preparative thin-layer chromatography were identified by ^1^H and ^13^C- NMR and compared with NMR obtained from standards (Figures S1 and S2, Supplementary Materials).

### 4.5. In Vivo Assays

#### 4.5.1. Animals

Female BALB/c mice 8 to 10 weeks of age and weighing 20 ± 5 g were used, obtained from the Bioterium of the National Medical Center “Siglo XXI” of the Mexican Institute of Social Security (IMSS). The animals were kept under standard laboratory conditions (temperature of 22 ± 2 °C, relative humidity of 50%, and light/dark cycle of 12 h) with standard feeding 5001 (Lab Diet^®^, Saint Louis, MO, USA) and purified water ad libitum. The experimental procedures were approved by the Ethics Committee of the Specialty Hospital of the National Medical Center “Siglo XXI” (IMSS), with registration numbers R-2023-3601-225 and R-2024-3601-203and were carried out in accordance with the Official Mexican Standards for the care and use of experimental animals [71].

#### 4.5.2. Acute Oral Toxicity

Acute oral toxicity was assessed in accordance with Organization for Economic Co-operation and Development (OECD) Guideline 423 for the evaluation of chemicals [72]. Female BALB/c mice were randomly distributed into three experimental groups (n = 3 per group). The treatments were administered orally in a single dose, through an esophageal cannula, after a period of fasting, with free access to water maintained throughout the procedure. The experimental groups included a healthy control (no treatment), a group treated only with the vehicle (Tween 80 at 2% in water), and a group that received the aqueous extract from the stem at doses of 3000 mg/kg.

After administration, the animals were monitored for the first 4 h to identify signs of toxicity, such as drowsiness, lethargy, seizures, tremors, or diarrhea, and possible mortality events. Follow-up lasted a period of 14 days.

Once the protocol was completed, the animals were sacrificed, and a macroscopic inspection of the main organs (liver, intestines, stomach, spleen, and kidneys) was carried out to detect morphological alterations attributable to toxicity. In addition, the weight of these organs was recorded, and the values obtained were compared with those of the control group. Based on these results, the mean lethal dose (LD_50_) for each compound was calculated, and its toxicity was classified according to the criteria of acute systemic toxicity proposed by the OECD, distributed in the following categories: category 1 (very toxic, ≤5 mg/kg); category 2 (toxic, >5 and ≤50 mg/kg); category 3 (harmful, >50 and ≤300 mg/kg); category 4 (low risk, >300 and ≤2000 mg/kg); and category 5 (unclassified, >2000 mg/kg).

#### 4.5.3. Experimental Type 2 Diabetes Induction

Experimental diabetes mellitus was induced using the streptozocin-induced type 2 (ST2D) model described above [30]. The mice were fasted for 16 h before the treatments were administered. Streptozocin (STZ) was dissolved in a 0.1 M cold buffer solution at pH 4; subsequently, the experimental treatment was administered at 100 mg/kg intraperitoneally (PI) on days 1 and 3. Nicotinamide (NA) was dissolved in cold saline and administered at 240 mg/kg PI, 30 min after STZ treatment, on day 1 only. At the end of treatment (day 3), the animals were offered a 10% sucrose solution ad libitum for two days. On day 5, the sucrose solution was removed and replaced with water ad libitum.

Two days after administration, the blood glucose of the animals was measured with blood samples obtained by puncture of the caudal vein with the glucose oxidase method, using test strips and a glucometer (ACCU-CHECK ^®^ Performa Blood Glucose Systems, Roche ^®^, DC, Basel, Switzerland). For subsequent testing, animals with glycemia between 290 and 390 mg/dL on the third day after STZ injection were considered diabetic and were used in this study. To confirm the experimental type 2 model of diabetes mellitus, a dose of 5 mg/kg of glibenclamide, a secretagogue drug, was administered orally to confirm the existence of functional β cells due to the action of streptozocin [73].

### 4.6. Grouping

For acute and subchronic evaluations, 24 groups were randomly formed (n = 6). The groups were as follows: aqueous extract of *A. cherimola* leaves (AEAcL) at doses of 100, 200, and 300 mg/kg, aqueous extract of *A. cherimola* stem (AEAcS) at doses of 100, 200, and 300 mg/kg.

Combinations of extracts and oral drugs: AEAcL + Met (100/850 mg/kg), AEAcL + Met (200/850 mg/kg), AEAcL + Met (100/500 mg/kg), AEAcL + Met (200/500 mg/kg), AEAcL + Aca (100/50 mg/kg), AEAcL + Gli (100/5 mg/kg), AEAcS + Met (100/500 mg/kg), AEAcS + Aca (100/50 mg/kg), AEAcS + Gli (100/5 mg/kg). For the flavonoid evaluation, the following groups were added: metformin (Met) 500 mg/kg, acarbose (Aca) 50 mg/kg, glibenclamide (Gli) 5 mg/kg, rutin 50 mg/kg, nicotiflorin 50 mg/kg, and narcissin 50 mg/kg. All samples were dissolved in 2%Tween 80, in water as a vehicle. The normoglycemic (healthy) and ST2D control groups received the vehicle; all treatments were administered orally through a gastric tube at 0.5 mL for each animal.

#### 4.6.1. Acute Evaluation of Aqueous Extracts from *Annona cherimola* and Its Combinations

The antihyperglycemic activity of animals with glycemia between 290–390 mg/dL, and with a positive response to glibenclamide, was determined, and they were considered type 2 diabetics. Once diabetic mice were identified, the AEAcL and AEAcS extracts were administered alone and in combination with oral antidiabetic drugs (metformin, acarbose, and glibenclamide) at the above-mentioned doses, intragastrical, with an esophageal cannula in a volume of 0.5 mL for each animal. Blood samples were obtained from the tail vein and blood glucose was measured with a conventional glucometer (ACCU-CHECK^®^ Performa Blood Glucose Systems, Roche^®^, DC, Basilea, Suiza); measurements were made at baseline (t = 0) and at 0.5, 1, 3, 5, and 7 h after administration of treatments.

#### 4.6.2. Subchronic Evaluation of Aqueous Leaf Extract of *Annona cherimola* in the ST2D Mouse Model

The same animals that were evaluated in the acute model were used; they received administrations daily with the treatments described above, in addition to having a weekly record of glycemic values kept with the help of a conventional glucometer for 12 weeks. In addition, the lipid profile, HbA1c, was monitored at weeks 0, 4, 8, and 12 of treatment.

#### 4.6.3. Measurement of % HbA1c

For the measurement of glycosylated hemoglobin (HbA1c), blood samples were collected from the caudal vein of the treated animals. The samples were analyzed using an automated boronate affinity system, using the Clover HbA1c Reader (Infopia^®^, Anyang, Republic of Korea), to determine the percentage of HbA1c in whole blood.

#### 4.6.4. Lipid Profile Measurement

To carry out the measurements, blood samples were obtained from the caudal vein of the animals. The analyses were carried out using the VERI-Q^®^ monitoring equipment, evaluating the following lipid parameters: total cholesterol (Chol), triglycerides (TG), high-density cholesterol (HDL-c), and low-density cholesterol (LDL-c).

#### 4.6.5. Oral Sucrose Tolerance Assay

The oral sucrose tolerance test (OSTT) was carried out following the protocol described by Calzada et al. [29], using fasting normoglycemic male mice. The animals were randomly assigned into six experimental groups (n = 6 per group): a control group that received Tween 80 at 2% in distilled water; a sucrose control group, treated with a vehicle plus a sucrose load (3 g/kg); three experimental groups that received the flavonoid rutin, nicotiflorin, or narcissin (50 mg/kg each); and a group treated with acarbose (50 mg/kg), used as a pharmacological control due to its inhibitory action on the enzyme α-glucosidase. All compounds were dissolved in the corresponding vehicle and administered orally via gastric cannula. Time zero (0 h) was established immediately before the administration of treatments. Thirty minutes later, the animals were given an oral load of sucrose (3 g/kg). Blood samples were collected at 1 and 2 h after carbohydrate administration, with glucose concentrations determined using the enzymatic glucose oxidase method.

#### 4.6.6. Oral Glucose Tolerance Assay

The oral glucose tolerance test (OGTT) was performed following the same procedure described for the oral sucrose tolerance test. However, in this evaluation, glucose (1.5 g/kg) was used as a carbohydrate source and canagliflozin (50 mg/kg) was used as a positive control, given its recognized hypoglycemic activity. Blood glucose concentrations were determined using the enzymatic glucose oxidase method, using the same experimental and sampling conditions described above.

### 4.7. Studies of Molecular Docking of Flavonoids

To perform molecular docking, flavonoids obtained from the pubchem database (https://pubchem.ncbi.nlm.nih.gov/, accessed on 10 June 2025)—rutin (CID: 5280805), nicotiflorin (CID:5318767), narcissin (CID: 5481663), acarbose (CID: 41774), canagliflozin (CID: 24812758), and 1YI (CID: 76070894)—were used. The ligands were optimized and subjected to energetic and geometric minimization using Avogadro software (version 1.2.0) [74]. Two targets were used. The first target was the enzyme α-glucosidase (crystal structure of the N-terminal of human maltase-glucoamylase, RCSB, PDB ID: 2QMJ), key in the digestion of oligosaccharides, determined by X-ray diffraction at 1.9 Å resolution in a complex with acarbose; this enzyme belongs to the GH31 family of glycosidases and has a catalytic domain type (β/α)_8_, where the active site is located. The second target was a SGLT1 (PDB ID: 7WMV) structure with a resolution of 3.2 Å and determined by electron microscopy; it is a sodium-dependent glucose cotransporter belonging to the sodium/solute transporter (SSF) superfamily, assembled with MAP17 (an accessory protein necessary for its correct assembly and activity). Both proteins were obtained from PDB (RCSB) (http://www.rcsb.org/, accessed on 12 June 2025).

The objectives were prepared to meet the conditions required in the study. In this process, water molecules not essential for the catalytic activity of the enzyme were removed. The sulfate ion was not removed, due to its importance in the catalytic function of α-glucosidase. All polar hydrogen atoms were protonated in a physiological environment (pH 7.4), and Gasteiger charges were assigned to the corresponding atoms. Finally, the resulting topologies were used as input files for the molecular docking simulations.

The in silico molecular docking experiments were carried out using AutoDock 4.2 software [75]. For the evaluation of the ligand–receptor interaction, the Lamarckian genetic algorithm was used as a scoring function, starting with a random population of 100 individuals and establishing a maximum number of energy evaluations of 1 × 10^7^ cycles. The grid box was defined with dimensions of 90 × 90 × 90 Å at each spatial coordinate, using a grid point spacing of 0.375 Å, ensuring adequate coverage of the active site for the docking simulations. Coupled complexes were visualized in Discovery Studio (BIOVIA, Dassault Systèmes, Discovery Studio Visualizer, 4.5, Dassault Systèmes, San Diego, CA, USA) and PyMOL (The PyMOL Molecular Graphics System, Ver 2.0, Schrödinger, LLC, DeLano Scientific, San Carlos, CA, USA). The validation of the molecular coupling studies was carried out by recoupling the cocrystallized ligand for each of the target proteins. The mean square deviation of atoms (RMSD) was calculated, being considered a reliable value when it was within the range of 2.2 Å. This calculation was carried out by superimposing the cocrystallized ligand with the lowest energy conformation obtained, evaluating whether it retained the same orientation and binding position in the active site of the protein, which ensures the reliability of the docking protocol. For the enzyme glucosidase, redocking with acarbose was performed and, for the SGLT1 ligand 1YI, the RMSDs obtained were 1.98 and 2.2, respectively.

### 4.8. In Silico Toxicology and Pharmaceutical Properties

Various computational tools were used to evaluate the pharmacokinetic, toxicological, and physicochemical properties of flavonoids, including Molinspiration [76], SwissADME [77], ADMETlab [78], and Tox-prediction [79]. These in silico predictors are based on the evaluation of the pharmacological nature and chemical compatibility with potential drugs of one or more small molecules, also allowing the identification and prioritization of candidate molecules with therapeutic potential in the development of new pharmacological agents.

### 4.9. Statistical Analysis

The results were expressed as mean ± standard error of mean (SEM). All statistical analyses were performed using GraphPad Prism version 8.2.1 (GraphPad Software Inc., San Diego, CA, USA). The statistical assessment was performed by an analysis of variance (ANOVA) followed by a Tukey test for multiple comparisons, considering that a *p*-value ≤ 0.05 represented a statistically significant difference.

## 5. Conclusions

The aqueous extract obtained from infusions of the leaves of *Annona cherimolla* Miller as a treatment only at the dose of 100 mg/kg and in combination with Met at the dose of 100/500 mg/kg obtained good antihyperglycemic and antihyperlipidemic activity in mice with experimental diabetes induced by streptozotocin in subchronic studies. The flavonoids rutin, nicotiflorin, and narcissin identified and isolated from the extract, confirmed that some of the pharmacological activities evaluated are due to these agents, although they have pharmacokinetic limitations that could be addressed with structural modifications or specific formulations. In addition, in silico and in vivo studies using OSTT and OGTT tests suggest that the antihyperglycemic activity of the three flavonoids could be mediated by the inhibition of SGLT1 and, particularly, that rutin and nicotinflorin act on the enzyme α-glucosidase. However, the mechanism of action of the flavonoids needs to be elucidated by other tests that confirm these findings.

## Figures and Tables

**Table 1:** Blood glucose levels of normoglycemic mice (healthy) and streptozocin type 2 diabetic mice (ST2D) at 0, 0.5, 1, 3, 5, and 7 h on the acute antihyperglycemic test.

Treatment	Glycemia (mg/dL)					
	0 h	0.5 h	1 h	3 h	5 h	7 h
Healthy	107 ± 2.4	124.6 ± 3.0 ^Δ^	129.8 ± 3.38 ^#^	124.8 ± 3.7 ^##^	123 ± 7.4 ^θ^	122.8 ± 5.5 ^θθ^
ST2D	349.8 ± 7.2	398 ± 32.4	413.6 ± 20.4 *	464 ± 21 *	491.8 ± 37.5 *	475.6 ± 38.9 *
AEAcL (100 mg/kg)	330.3 ± 21.1	429.6 ± 33.1 *	384.3 ± 37.1	362 ± 32.6 ^##^	372 ± 40.5 ^θ^	330 ± 43.9 ^θθ^
AEAcL (200 mg/kg)	307 ± 16.4	288.3 ± 21.4 ^Δ^	286.6 ± 22.1 ^#^	273.3 ± 31.2 ^##^	248 ± 17.1 *^θ^	263 ± 14.9 ^θθ^
AEAcL (300 mg/kg)	324.4 ± 33.6	348.2 ± 25.4	350.4 ± 20.5	288 ± 14.5 ^##^	294.4 ± 23.6 ^θ^	315.6 ± 20.8 ^θθ^
AEAcS (100 mg/Kg)	299.7 ± 5	400.7 ± 20.1 *	367 ± 27.9 *	333.3 ± 33.7 ^##^	361.6 ± 35.3 *^θ^	372.4 ± 32.6 *^θθ^
AEAcS (200 mg/Kg)	329 ± 12.4	324.4.6 ± 34.9	297.7 ± 31.7 ^#^	282 ± 40.3 ^##^	275.6 ± 33.5 ^θ^	270.8 ± 34.7 *^θθ^
AEAcS (300 mg/Kg)	340.4 ± 12.2	404.04 ± 19.4	276.8 ± 23.1 ^#^	368 ± 6 ^##^	355.5 ± 6.9 ^θ^	267.5 ± 12.6 *^θθ^
AEAcL + Met (100/850 mg/kg)	313.3 ± 12	144 ± 26 *^Δ^	110 ± 21.2 *^#^	68 ± 5.2 *^##^	73.3 ± 3.7 *^θ^	102.3 ± 2.5 *^θθ^
AEAcL + Met (200/850 mg/kg)	351 ± 22.6	366 ± 26.6	287.5 ± 21.1 *^#^	179 ± 11.6 *^##^	70.5 ± 4.8 *^θ^	80 ± 5 *^θθ^
AEAcL + Met (100/500 mg/kg)	346 ± 15.4	357.6 ± 22	240.8 ± 36.2 *^#^	164.4 ± 35.5 *^##^	143.2 ± 29.1 *^θ^	161.2 ± 38.6 *^θθ^
AEAcL + Met (200/500 mg/kg)	356.3 ± 7.3	146.6 ± 24.2 *^Δ^	109.6 ± 11 *^#^	67.6 ± 18.4 *^##^	48.6 ± 9.7 *^θ^	113.3 ± 35.6 *^θθ^
AEAcL + Aca(100/50 mg/kg)	382.7 ± 4.7	333.5 ± 21.8 *	300.2 ± 21.3 *^#^	294.7 ± 22.3 *^##^	338.75 ± 26 *^θ^	396 ± 34.9
AEAcL + Gli(100/5 mg/kg)	334.3 ± 12.8	454.6 ± 23.3 *	464.3 ± 16.8 *	402.3 ± 8.6 *^##^	364.6 ± 6.9 ^θ^	399.3 ± 19.4 *
AEAcS + Met (100/500 mg/kg)	331.75 ± 14	293.75 ± 19.7 ^Δ^	178.5 ± 17.2 *^#^	65.75 ± 10.1 *^##^	151.25 ± 31 *^θ^	241.25 ± 34.5 *^θθ^
AEAcS + Aca(100/50 mg/kg)	375.6 ± 2.73	298.3 ± 5.9 *^Δ^	329.6 ± 3.7 *^#^	311 ± 11 *^##^	332.6 ± 4.3 *^θ^	336.6 ± 4.2 ^θθ^
AEAcS + Gli(100/5 mg/kg)	350.3 ± 13	433.3 ± 10.3 *	404.6 ± 2.6 *	383.6 ± 12.8	386.3 ± 21.5 ^θ^	387.6 ± 17.5

Data are expressed as means ± SEM, n = 6. * *p* < 0.05 vs. initial values; ^Δ^ *p* < 0.05 vs. ST2D 0.5 h; ^#^ *p* < 0.05 vs. ST2D 1 h; ^##^ *p* < 0.05 vs. ST2D 3 h; ^θ^ *p* < 0.05 vs. ST2D 5 h; ^θθ^ *p* < 0.05 vs. ST2D 7 h. ST2D: streptozocin-induced type 2 diabetic group; AEAcL: aqueous extract from *A. cherimola* leaves; AEAcS: aqueous extract from *A. cherimola* stem; Met: metformin; Aca: acarbose; Gli: glibenclamide.

**Table 2:** Lipid profile values (cholesterol, triglycerides, HDL-c, and LDL-c) at the beginning (0), 4, 8, and 12 weeks of subchronical assay.

Lipid Profile								
**Cholesterol (mg/dL)**					**Triglycerides (mg/dL)**			
**Treatment**	**Week**				**Week**			
	**0**	**4**	**8**	**12**	**0**	**4**	**8**	**12**
Healthy	97.6 ± 0.8	99 ± 1	99.6 ± 1.7	100.3 ± 0.8	69.6 ± 4.7	73 ± 1	72 ± 2.3	71.3 ± 0.8
ST2D	101.3 ± 1.2	100.6 ± 1.2	101.3 ± 1.4	104 ± 2	68 ± 1.1	125 ± 1.1 ^#^	138 ± 10 ^#^	140 ± 4 ^#^
AEAcL	100.6 ± 1.2	96.6 ± 1.4	105 ± 1.1	101 ± 1.1	75.3 ± 2.3	89.6 ± 4.1 ^#^*	87.3 ± 2 ^#^*	92.6 ± 1.4 ^#^*
Met	101.3 ± 1.2	113.3 ± 5.4 ^#^*	101.3 ± 0.8	100.3 ± 0.8	68 ± 1.1	156 ± 6 ^#^*	146 ± 2 ^#^	156 ± 1.1 ^#^*
AEAcL + Met	100.6 ± 1.2	102.3 ± 1.7	103.3 ± 3.5 *	102 ± 1.7	68 ± 1.1	83.3 ± 2.4 *	85.3 ± 2.1 *	76.6 ± 1.2 *
	
**HDL-c (mg/dL)**					**LDL-c (mg/dL)**			
**Treatment**	**Week**				**Week**			
	**0**	**4**	**8**	**12**	**0**	**4**	**8**	**12**
Healthy	78.7 ± 3.5	65 ± 5.7	65.3	63.7	53 ± 1.7	46 ± 1.7	52 ± 4	64.3 ± 2 *
ST2D	80 ± 1.1	27.3 ± 1.2 ^#^	33.6 ± 2 ^#^	23 ± 4 ^#^	56.1 ± 1.4 ^#^	100 ± 1.5 ^#^	86 ± 1.1 ^#^	100 ± 1.5 ^#^
AEAcL	77.6 ± 1.3	61 ± 3 *	66 ± 6.4 *	72.3 ± 1.4 *	55 ± 2.6 *	48 ± 1.1 *	49 ± 3.4 *	65 ± 5.6 *
Met	78.6 ± 4.3	81.3 ± 1.2 ^#*^	80 ± 2.3 ^#^*	82.3 ± 1.6 ^#^*	58.1 ± 2.6 ^#^	108.6 ± 8.7 ^#^	105.6 ± 1.4 ^#^*	102 ± 1.1 ^#^
AEAcL + Met	79 ± 6.2	67 ± 1.1 *	67 ± 1.1 *	65 ± 1.1 *	57 ± 2.5 *	55.6 ± 1.7 *	60 ± 8 *	65 ± 2.6 *

Results expressed as the mean ± SEM, n = 6. ^#^ *p* < 0.05 vs. healthy control; * *p* < 0.05 vs. ST2D control. T2D: streptozocin-induced type 2 diabetic group; AEAcL: aqueous extract from *A. cherimola* leaves 100 mg/kg; Met: metformin 500 mg/kg; AEAcL + Met: combination of aqueous extract from *A. cherimola* leaves + metformin 100/500 mg/kg.

**Table 3:** Blood glucose levels of normoglycemic mice (healthy) and type 2 diabetic mice induced with streptozocin (ST2D) at 0, 2, and 4 h, in the acute antihyperglycemic test.

Treatment	Glycemia (mg/dL)		
	0	2	4
Healthy	145 ± 0.6 ^Δ^	149 ± 9	150.2 ± 1.6
ST2D	340.4 ± 6.5	474 ± 33.6 *	477.3 ± 29 *
AEAcL	329 ± 9	373.1 ± 28.7 ^#^	367 ± 31.6 ^##^
Met	361.9 ± 7.6	384.5 ± 1.6 *^#^	371.4 ± 2.33 ^##^
Aca	346.5 ± 3.9	329.5 ± 10 ^#^	275 ± 11 *^##^
Gli	348.1 ± 11	267.1 ± 0.32 *^#^	353.2 ± 0.4 ^##^
Rutin	321.2 ± 3	185 ± 6.4 *^#^	168.5 ± 2.9 *^##^
Nicotiflorin	307.3 ± 0.28	180.4 ± 1.6 *^#^	185 ± 2 *^##^
Narcissin	311.6 ± 3	188 ± 2 *^#^	178.2 ± 5 *^##^

Data are expressed as means ± SEM, n = 6. * *p* < 0.05 vs. initial values; ^Δ^ *p* < 0.05 vs. ST2D 0 h; *^#^ p* < 0.05 vs. ST2D 2 h; *^##^ p* < 0.05 vs. ST2D 4 h. ST2D: streptozocin-induced type 2 diabetic group; AEAcL: aqueous extract from *A. cherimola* leaves 100 mg/kg; Met: metformin 500 mg/kg; Aca: acarbose 50 mg/kg; Gli: glibenclamide 5 mg/kg.

**Table 4:** Binding energies and interactions of rutin, nicotiflorin, and narcissin on α-glucosidase and SGLT1.

Compound	α-Glucosidase		
	ΔG (kcal/mol)	H-Binding Residues	NPI
Rutin	−4.44	Aps 203, Thr 204, Thr 205, Tyr 299, Asp 327, Ile 364, Trp 441, Ser 448, Phe 450, Lys 480, Arg 526, Trp 539, Asp 542, Ala 576, Leu 577, His 600, Gly 602, Gln 603, Tyr 605	Trp 406, Met 444, Phe 575
Nicotiflorin	−5.23	Asp 203, Thr 205, Pro 206, Tyr 299, Asp 327, Asp 366, Trp 441, Asp 443, Met 444, Arg 526, Asp 542, Asn 543, Thr 544, Thr 544, His 600, Gln 603, Tyr 605	Ile 328, Ile 364, Trp 406, Phe 575, Ala 576
Narcissin	−5.61	Asp 203, Thr 204, Tyr 299, Asp 327, Asp 366, Trp 441, Asp 443, Met 444, Arg 526, Asp 542, His 600, Gly 602, Gln 603	Ile 328, Ile 364, Trp 406, Phe 450, Phe 575, Ala 576, Tyr 605
Acarbose	−4.36	Asp 203, Thr 204, Thr 205, Asn 207, Asp 327, Ile 328, Ile 364, Trp 441, Asp 443, Met 444, Lys 480, Arg 526, Trp 539, Gly 541, Asp 542, Thr 544, Ala 576, Arg 598, His 600	Tyr 299, Trp 406, Phe 575
**Compound**	**Sodium–Glucose Cotransporter (SGLT1)**		
Rutin	24.12	Phe 251, Asp 273, Cys 345, Val 346, Pro 348, Val 359, Gly 360, Thr 362, Asn 363, Gly 450, Gln 451, Asp 454, Gly 509, Ser 510, Met 512, His 525, Tyr 526	Cys 255, Cys 351, Cys 511
Nicotiflorin	−2.15	Thr 90, Ala 93, Ser 94, Lys 254, Cys 255, Tyr 256, Asp 273, Ala 344, Cys 345, Val 346, Cys 351, Val 359, Gly 360, Cys 361, Thr 362, Gly 450, Gln 451, Asp 454, Leu 274, Tyr 526	His 525
Narcissin	−1.17	Gly 82, His 83, Gly 86, Leu 87, Thr 90, Ser 94, Ala 97, Asp 273, Leu 274, Thr 362, Gln 451, Asp 454, Gln 457, Tyr 526	Ala 93, Ile 98, Phe 101, Phe 453
Canagliflozin	−6.77	Gly 82, His 83, Leu 87, Glu 102, Thr 156, Met 283, Thr 287, Tyr 290, Trp 291, Lys 321, Phe 453, Gln 457, Thr 460	Ala 105, Lys 157, Aala 160, Leu 286, Trp 289, Ile 456

ΔG: Binding energy (kcal/mol); NPI: Non-polar interactions; Phe: Phenylalanine; Asp: Aspartate; Val: Valine; Ala: Alanine; Asn: Asparagine; Arg: Arginine; Cys: Cysteine; Tyr: Tyrosine; Thr: Threonine; Ser: Serine; Lys: Lysine; Met: Methionine; Trp: Tryptophan; Gly: Glycine; Leu: Leucine; Glu: Glutamic acid; Ile: Isoleucine; Pro: Proline; His: Histidine; Gln: Glutamine.

**Table 5:** Physicohemical, pharmacokinetic, and toxicologic predictive values of flavonoids ^a^.

	Rutin	Nicotiflorin	Narcissin		Rutin	Nicotiflorin	Narcissin
Physicochemical				Pharmacokinetics			
TPSA	269.43	249.2	258.4	Human intestinal absorption	Medium	Excellent	Excellent
Lipophilicity (logP)	0.98	1.16	0.72	BBBp	No	No	No
Water solubility (logS)	−2.39	−2.55	−2.64	Volume of distribution	0.87	0.91	0.79
Rotatable bonds	6	6	7	Plasma protein binding	85.0%	85.1%	84.5%
Number of H donors	10	9	9	CYP1A2 inhibitor	No	No	No
Number of H-bond acceptors	16	15	16	CYP2C19 inhibitor	No	No	No
Druglikeness				CYP2C19 substrate	No	No	No
Lipinski	No, 4 violations: MW > 480, WLogP < −0.4, MR > 130, #atoms > 70	No, 4 violations: MW > 480, WLogP < −0.4, MR > 130, #atoms > 70	No 4 violations: MW > 480, WLogP < −0.4, MR > 130, #atoms > 70	CYP2C9 inhibitor	No	No	No
				CYP2D6 inhibitor	No	No	No
				CYP2D6 substrate	No	No	No
Ghose	No 4 violations: MW > 480, WLogP < −0.4, MR > 130, #atoms > 70	No 4 violations: MW > 480, WLogP < −0.4, MR > 130, #atoms > 70	No 4 violations: MW > 480, WLogP < −0.4, MR > 130, #atoms > 70	CYP3A4 inhibitor	No	No	No
				Clearance	Low	Low	Low
				T1/2	4.6	4.27	4.34
Veber	No; 1 violation: TPSA > 140	No; 1 violation: TPSA > 140	No; 1 violation: TPSA > 140	Toxicity			
Egan	No; 1 violation: TPSA > 131.6	No; 1 violation: TPSA > 131.6	No; 1 violation: TPSA > 131.6	Mutagenic	No	No	No
				Carcinogenic	No	No	No
				Neurotoxicity	No	No	No
				Rat oral acute toxicity	No	No	No
				H-HT	Low	Low	Low
				Predicted toxicity class ^b^	5	5	5

Logarithm of the n-octanol/water distribution coefficient (logP), topological polar surface area (TPSA), human hepatotoxicity (H-HT), blood–brain barrier permeability (BBBp), molar refractivity (MR). ^a^ The predictions were obtained from bioinformatics tools Tox-prediction, ADMET*lab*, and SwissADME web servers, and they were ^b^ Class I: fatal if swallowed (LD_50_ ≤ 5), Class II: fatal if swallowed (5 < LD_50_ ≤ 50), Class III: toxic if swallowed (50 < LD_50_ ≤ 300), Class IV: harmful if swallowed (300 < LD_50_ ≤ 2000), Class V: may be harmful if ingested (2000 < LD_50_ ≤ 5000), Class VI: non-toxic (LD_50_ > 5000).

## Data Availability

The original contributions presented in this study are included in the article/Supplementary Material. Further inquiries can be directed to the corresponding authors.

## Supplementary Materials

The following supporting information can be downloaded at https://www.mdpi.com/article/10.3390/ph18111754/s1, Figure S1. ^13^C-NMR spectra of flavonoids (A) Rutin, (B) Nicotinflorin, (C) Narcissin. Figure S2. 1H-NMR spectra of flavonoids (A) Rutin, (B) Nicotinflorin, (C) Narcissin.

## References

[1] World Health Organization. https://www.who.int/es/news-room/fact-sheets/detail/diabetes.

[2] Instituto Nacional de Estadística y Geografía. https://www.inegi.org.mx/contenidos/saladeprensa/boletines/2025/edr/edr2024_en-jun_RR.pdf.

[3] Zhong J. (2020). Classification and diagnosis of diabetes: Standards of medical care in diabetes—2020. Diabetes Care.

[4] Galicia-Garcia U., Benito-Vicente A., Jebari S., Larrea-Sebal A., Siddiqi H., Uribe K.B., Martín C. (2020). Pathophysiology of type 2 diabetes mellitus. Int. J. Mol. Sci..

[5] DeFronzo R.A. (2009). From the triumvirate to the ominous octet: A new paradigm for the treatment of type 2 diabetes mellitus. Diabetes.

[6] Bhatti J.S., Sehrawat A., Mishra J., Sidhu I.S., Navik U., Khullar N., Reddy P.H. (2022). Oxidative stress in the pathophysiology of type 2 diabetes and related complications: Current therapeutics strategies and future perspectives. Free Rad. Biol. Med..

[7] Gudoor R., Suits A., Shubrook J.H. (2023). Perfecting the Puzzle of Pathophysiology: Exploring Combination Therapy in the Treatment of Type 2 Diabetes. Diabetology.

[8] Yubero-Serrano E.M., Gutiérrez-Mariscal F.M., Gómez-Luna P., Alcalá-Diaz J.F., Pérez-Martinez P., López-Miranda J. (2023). Dietary modulation of advanced glycation end products metabolism on carotid intima-media thickness in type 2 diabetes patients: From the CORDIOPREV study. Clín. Investig. Arterioscler..

[9] Singh V.P., Bali A., Singh N., Jaggi A.S. (2014). Advanced glycation end products and diabetic complications. Korean J. Physiol. Pharmacol..

[10] Kumar P.R., Bhansali A., Ravikiran M., Bhansali S., Dutta P., Thakur J.S., Walia R. (2010). Utility of glycated hemoglobin in diagnosing type 2 diabetes mellitus: A community-based study. J. Clin. Endocrinol. Metab..

[11] Dilworth L., Facey A., Omoruyi F. (2021). Diabetes mellitus and its metabolic complications: The role of adipose tissues. Int. J. Mol. Sci..

[12] Erion D.M., Park H.J., Lee H.Y. (2016). The role of lipids in the pathogenesis and treatment of type 2 diabetes and associated co-morbidities. BMB Rep..

[13] Weinstein A.R., Sesso H.D., Lee I.M., Cook N.R., Manson J.E., Buring J.E., Gaziano J.M. (2004). Relationship of physical activity vs body mass index with type 2 diabetes in women. JAMA.

[14] Venkatasamy V.V., Pericherla S., Manthuruthil S., Mishra S., Hanno R. (2013). Effect of Physical activity on Insulin Resistance, Inflammation and Oxidative Stress in Diabetes Mellitus. J. Clin. Diagn. Res..

[15] Fuchsberger C., Flannick J., Teslovich T.M., Mahajan A., Agarwala V., Gaulton K.J., Ma C., Fontanillas P., Moutsianas L., McCarthy D.J. (2016). The genetic architecture of type 2 diabetes. Nature.

[16] Flannick J., Florez J.C. (2016). Type 2 diabetes: Genetic data sharing to advance complex disease research. Nat. Rev. Genet..

[17] Barrientos-Ávalos J., Morel-Cerda E., Félix-Téllez F., Vidrio-Huerta B.E., Aceves-Ayala A.R., Flores-Rendón Á.R., Velarde-Ruiz Velasco J.A. (2024). Gastrointestial adverse effects of old and new antidiabetics: How do we deal with them in real life?. Rev. Gastroenterol. Mex. (Engl. Ed.).

[18] Soccio R.E., Chen E.R., Lazar M.A. (2014). Thiazolidinediones and the promise of insulin sensitization in type 2 diabetes. Cell Metab..

[19] Kumar S., Mittal A., Babu D., Mittal A. (2021). Herbal medicines for diabetes management and its secondary complications. Curr. Diabetes Rev..

[20] Chaachouay N., Zidane L. (2024). Plant-derived natural products: A source for drug discovery and development. Drugs Drug Candidates.

[21] Al Kazman B.S., Harnett J.E., Hanrahan J.R. (2022). Traditional uses, phytochemistry and pharmacological activities of annonacae. Molecules.

[22] Mannino G., Gentile C., Porcu A., Agliassa C., Caradonna F., Bertea C.M. (2020). Chemical profile and biological activity of cherimoya (*Annona cherimola* Mill.) and atemoya (*Annona atemoya*) leaves. Molecules.

[23] Vasarri M., Barletta E., Vinci S., Ramazzotti M., Francesconi A., Manetti F., Degl’Innocenti D. (2020). *Annona cherimola* Miller fruit as a promising candidate against diabetic complications: An in vitro study and preliminary clinical results. Foods.

[24] Quílez A.M., Fernández-Arche M.A., García-Giménez M.D., De la Puerta R. (2018). Potential therapeutic applications of the genus Annona: Local and traditional uses and pharmacology. J. Ethnopharmacol..

[25] Martínez-Vázquez M., Estrada-Reyes R., Araujo A. (2012). Antidepressant-like effects of an alkaloid extract of the aerial parts of *Annona cherimolia* in mice. J. Ethnopharmacol..

[26] Ammoury C., Younes M., El-Khoury M. (2019). The pro-apoptotic effect of a Terpene-rich *Annona cherimola* leaf extract on leukemic cell lines. BMC Complement. Altern. Med..

[27] Verma A., Kumar A., Shekar R., Kumar K., Chakrapani R. (2011). Pharmacological Screening of *Annona cherimola* for Antihyperlipidemic Potential. J. Basic. Clin. Pharm..

[28] Díaz-de-Cerio E., Aguilera-Saez L.M., Gómez-Caravaca A.M., Verardo V., Fernández-Gutiérrez A., Fernández I., Arráez-Román D. (2018). Characterization of bioactive compounds of *Annona cherimola* L. leaves using a combined approach based on HPLC-ESI-TOF-MS and NMR. Anal. Bioanal. Chem..

[29] Calzada F., Solares-Pascasio J.I., Ordoñez-Razo R.M., Velazquez C., Barbosa E., García-Hernández N., Correa-Basurto J. (2017). Antihyperglycemic activity of the leaves from *Annona cherimola* miller and rutin on alloxan-induced diabetic rats. Pharmacogn. Res..

[30] Martínez-Solís J., Calzada F., Barbosa E., Valdés M. (2021). Antihyperglycemic and antilipidemic properties of a tea infusion of the leaves from *Annona cherimola* miller on streptozocin-induced type 2 diabetic mice. Molecules.

[31] Martínez-Solís J., Calzada F., Barbosa E., Gutiérrez-Meza J.M. (2022). Antidiabetic and Toxicological Effects of the Tea Infusion of Summer Collection from *Annona cherimola* Miller Leaves. Plants.

[32] Valdes M., Calzada F., Martínez-Solís J., Martínez-Rodríguez J. (2023). Antihyperglycemic effects of *Annona cherimola* miller and the flavonoid rutin in combination with oral antidiabetic drugs on streptozocin-induced diabetic mice. Pharmaceuticals.

[33] Calzada F., Valdes M., Martínez-Solís J., Velázquez C., Barbosa E. (2023). *Annona cherimola* Miller and Its Flavonoids, an Important Source of Products for the Treatment of Diabetes Mellitus: In Vivo and In Silico Evaluations. Pharmaceuticals.

[34] Perrone A., Yousefi S., Salami A., Papini A., Martinelli F. (2022). Botanical, genetic, phytochemical and pharmaceutical aspects of *Annona cherimola* Mill. Sci. Hortic..

[35] Hedrington M.S., Davis S.N. (2019). Considerations when using alpha-glucosidase inhibitors in the treatment of type 2 diabetes. Expert. Opin. Pharmacother..

[36] Zhao M., Li N., Zhou H. (2023). SGLT1: A potential drug target for cardiovascular disease. Drug Des. Dev. Ther..

[37] Banday M.Z., Sameer A.S., Nissar S. (2020). Pathophysiology of diabetes: An overview. Avicenna J. Med..

[38] Schuster D.P., Duvuuri V. (2002). Diabetes mellitus. Clin. Podiatr. Med. Surg..

[39] Weinberg Sibony R., Segev O., Dor S., Raz I. (2023). Drug therapies for diabetes. Inter. J. Mol. Sci..

[40] Kane J.P., Pullinger C.R., Goldfine I.D., Malloy M.J. (2021). Dyslipidemia and diabetes mellitus: Role of lipoprotein species and interrelated pathways of lipid metabolism in diabetes mellitus. Curr. Opin. Pharm..

[41] Salehi B., Ata A., Anil Kumar N.V., Sharopov F., Ramirez-Alarcon K., Ruiz-Ortega A., Ayatollahi S.A., Fokou P.V.T., Kobarfard F., Zakaria Z.A. (2019). Antidiabetic potential of medicinal plants and their active components. Biomolecules.

[42] Encuesta Nacional de Salud y Nutrición. https://ensanut.insp.mx..

[43] Arumugam G., Manjula P., Paari N. (2013). A review: Anti diabetic medicinal plants used for diabetes mellitus. J. Acute Dis..

[44] Yi X., Pan Y., Peng H., Ren M., Jia Q., Wang B. (2024). The optimal dose of metformin to control conversion to diabetes in patients with prediabetes: A meta-analysis. J. Diabetes Complicat..

[45] Tian J., Li C., Dong Z., Yang Y., Xing J., Yu P., Xin Y., Xu F., Wang L., Mu Y. (2023). Inactivation of the antidiabetic drug acarbose by human intestinal microbial-mediated degradation. Nat. Metab..

[46] Hadi F., Sardar H., Al-Otaibi J.S., Pirzada A.S., Alam W., Khan H. (2025). Comprehensive in vivo Antidiabetic Investigations and antioxidant potential of Caralluma edulis whole Plant. Curr. Res. Nutr. Food Sci. J..

[47] Gupta R.C., Chang D., Nammi S., Bensoussan A., Bilinski K., Roufogalis B.D. (2017). Interactions between antidiabetic drugs and herbs: An overview of mechanisms of action and clinical implications. Diabetol. Metab. Syndr..

[48] Khalid M., Petroianu G., Adem A. (2022). Advanced glycation end products and diabetes mellitus: Mechanisms and perspectives. Biomolecules.

[49] Falé P.L., Ferreira C., Maruzzella F., Florêncio M.H., Frazão F.N., Serralheiro M.L. (2013). Evaluation of cholesterol absorption and biosynthesis by decoctions of *Annona cherimola* leaves. J. Ethnopharmacol..

[50] Vinayagam R., Xu B. (2015). Antidiabetic properties of dietary flavonoids: A cellular mechanism review. Nutr. Met..

[51] Hanis N., Ismail N.A., Ali E.Z. (2025). Systematic review on effectiveness of flavonoids against hypercholesterolemia: Insights from in-silico, in-vitro, and in-vivo studies. Food Chem. Adv..

[52] Ahmed O.M., Moneim A.A., Yazid I.A., Mahmoud A.M. (2010). Antihyperglycemic, antihyperlipidemic and antioxidant effects and the probable mechanisms of action of Ruta graveolens infusion and rutin in nicotinamide-streptozotocin-induced diabetic rats. Diabetol. Croat..

[53] Ghorbani A. (2017). Mechanisms of antidiabetic effects of flavonoid rutin. Biomed. Pharmacother..

[54] Tahsin M.R., Tithi T.I., Mim S.R., Haque E., Sultana A., Bahar N.B., Amran M.S. (2022). In vivo and in silico assessment of diabetes ameliorating potentiality and safety profile of Gynura procumbens leaves. Evid.-Based Complement. Altern. Med..

[55] Lam T.-P., Tran N.-V.N., Pham L.-H.D., Lai N.V.-T., Dang B.-T.N., Truong N.-L.N., Nguyen-Vo S.-K., Hoang T.-L., Mai T.T., Tran T.-D. (2024). Flavonoids as dual-target inhibitors against α-glucosidase and α-amylase: A systematic review of in vitro studies. Nat. Prod. Bioprospecting.

[56] Yin Z., Zhang W., Feng F., Zhang Y., Kang W. (2014). α-Glucosidase inhibitors isolated from medicinal plants. Food Sci. Hum. Wellness.

[57] Limanto A., Simamora A., Santoso A.W., Timotius K.H. (2019). Antioxidant, α-glucosidase inhibitory activity and molecular docking study of gallic acid, quercetin and rutin: A comparative study. Mol. Cell Biomed. Sci..

[58] Foo S., Chan S., Sharryl T., Puah J., Hong Y., Thibiya D., Baskaram G., Shamala S. (2021). Hypoglycemic effects od plant flavonoids: A Review. Evid.-Based Complement. Altern. Med..

[59] Pham A., Malterud K., Paulsen B., Diallo D., Wangensteen H. (2014). α-Glucosidase inhibition, 15-lipoxygenase inhibition, and brine shrimp toxicity of extracts and isolated compounds from *Terminalia macroptera* leaves. Pharm. Biol..

[60] Li Y.Q., Zhou F.C., Gao F., Bian J.S., Shan F. (2009). Comparative evaluation of quercetin, isoquercetin and rutin as inhibitors of α-glucosidase. J. Agric. Food Chem..

[61] Pires D.E., Blundell T.L., Ascher D.B. (2015). pkCSM: Predicting small-molecule pharmacokinetic and toxicity properties using graph-based signatures. J. Med. Chem..

[62] Yang C.Y., Hsiu S.L., Wen K.C., Lin S.P., Tsai S.Y., Hou Y.C., Chao P.D. (2005). Bioavailability and metabolic pharmacokinetics of rutin and quercetin in rats. J. Food Drug Anal..

[63] Wang Y.N., Jin H., Fan H.R., Wang B.L. (2024). Simultaneous assessment of absorption and pharmacokinetic characteristics of four active flavonoids from Chimonanthus nitens Leaf Granules using LC-MS determination: In vivo and in vitro. J. Asian Nat. Prod. Res..

[64] Pollastri M.P. (2010). Overview on the Rule of Five. Curr. Prot. Pharmacol..

[65] Park M.J., Kang Y.H. (2020). Isolation of Isocoumarins and Flavonoids as α-Glucosidase Inhibitors from *Agrimonia pilosa* L. Molecules.

[66] Zhu H., Zhong X. (2022). Synthesis of activity evaluation of flavonoid derivatives as ɑ-glucosidase inhibitors. Front. Chem..

[67] Yi X., Dong M., Guo N., Tian J., Lei P., Wang S., Yang Y., Shi Y. (2023). Flavonoids improve type 2 diabetes mellitus and its complications: A review. Front. Nutr..

[68] Shamsudin N.F., Ahmed Q.U., Mahmood S., Shah S.A.A., Sarian M.N., Khattak M.M.A.K., Khatib A., Sabere A.S.M., Yusoff Y.M., Latip J. (2022). Flavonoids as Antidiabetic and Anti-Inflammatory Agents: A Review on Structural Activity Relationship-Based Studies and Meta-Analysis. Int. J. Mol. Sci..

[69] Dunya A., Duhaidahawi S., Haider F. (2021). Flavonoids in the Treatment of Diabetes: Clinical Outcomes and Mechanism to Ameliorate Blood Glucose Levels. Curr. Diabetes Rev..

[70] Abdelhakim B., Abdelaali B., Asaad K., Hafiz A., Hassan A., Mohamed A., Hermansyah A., Long C., Khang W., Nasreddine E. (2024). Clinical applications and mechanism insights of natural flavonoids against type 2 diabetes mellitus. Heliyon.

[71] Norma Oficial Mexicana (1999). NOM-062-ZOO-1999: Especificaciones Técnicas Para la Producción, Cuidado y Uso de Los Animales de Laboratorio. https://www.fmvz.unam.mx/fmvz/principal/archivos/062ZOO.PDF.

[72] OECD (2001). Guideline for Testing of Chemicals 423. Acute Oral Toxicity-Acute Toxic Class Method. Organización Para la Cooperación y el Desarrollo Economicos, OECD/OCDE. https://www.oecd.org/chemicalsafety/risk-assessment/1948378.pdf.

[73] Hsu J., Wu C., Hung C., Wang C., Huang H. (2016). Myrciaria cauliflora extract improves diabetic nephropathy via suppression of oxidative stress and inflammation in streptozotocin-nicotinamide mice. J. Food Drug Anal..

[74] Hanwell M., Curtis D., Lonie D., Vandermeersch T., Zurek E., Hutchison G. (2012). Avogadro: An advanced semantic chemical editor, visualization, and analysis platform. J. Cheminform..

[75] Morris G., Lindstrom W., Sanner M., Belew R., Goodshell D., Olson A. (2009). Autodock4 and AutodockTools4: Automated docking with selective receptor flexibility. J. Comput. Chem..

[76] Molinspiration Cheminformatics 2017 Calculation of Molecular Properties and Bioactivity Score. http://www.molinspiration.com/services/logp.html..

[77] Daina A., Michielin O., Zoete V. (2017). SwissADME: A free web tool to evaluate pharmacokinetics, drug-likeness and medicinal chemistry friendliness of small molecules. Sci. Rep..

[78] Xiong G., Wu Z., Yi J., Fu L., Yang Z., Hsieh C., Cao D. (2021). ADMETlab 2.0: An integrated online platform for accurate and comprehensive predictions of ADMET properties. Nucleic Acids Res..

[79] Banerjee P., Kemmler E., Dunkel M., Preissner R. (2024). ProTox 3.0: A webserver for the prediction of toxicity of chemicals. Nucleic Acids Res..

