# Supplementary material for: Aqueous Extracts and Flavonoids Obtained from Annona cherimola Miller as Antidiabetic Treatments Alone and in Combination with Antidiabetic Drugs: In Vivo and In Silico Studies
Source: Pharmaceuticals (Basel). 2025 Nov 18;18(11):1754. doi: 10.3390/ph18111754 (PMC12655703; doi:10.3390/ph18111754)
Supplement: Supplementary file 1 [file pharmaceuticals-18-01754-s001.zip › pharmaceuticals-3986178-supplementary.pdf]

Aqueous extracts and flavonoids obtained from *Annona cherimola* Miller as antidiabetic treatments alone and in combination with antidiabetic drugs: In vivo and In silico studies, Part I

Jesica Ramírez-Santos [1*], Fernando Calzada [1,*], Julita Martínez-Rodríguez [1], Miguel Valdes [1,2,3], Elizabeth Barbosa [2] and Claudia Velázquez [3]

[1] Unidad de Investigación Médica en Farmacología, UMAE Hospital de Especialidades, Centro Médico Nacional Siglo XXI, Instituto Mexicano del Seguro Social, Mexico City CP 06720, Mexico;

[2] Instituto Politécnico Nacional, Sección de Estudios de Posgrado e Investigación, Escuela Superior de Medicina, Plan de San Luis y Salvador Díaz Mirón S/N, Col. Casco de Santo Tomás, Miguel Hidalgo, Mexico City CP 11340, Mexico; rebc78@yahoo.com.mx

[3] Laboratorio de Inmunología, Departamento de Sistemas Biológicos, Universidad Autónoma Metropolitana, Unidad Xochimilco, Calz. Del Hueso 1100, Col. Villa Quietud, Coyoacán, CP04960, Ciudad de México, México; valdesguevaramiguel@gmail.com

[4] Área Académica de Farmacia, Instituto de Ciencias de la Salud, Universidad Autónoma del Estado de Hidalgo, San Agustin Tlaxiaca CP 42076, Mexico; cvg09@yahoo.com

* Correspondence: fercalber10@gmail.com (F.C.); jes.ram.san@gmail.com (J.R.-S.)

Contents

Figure S1. ^{13}C-NMR spectra of flavonoids **A**) Rutin, **B**) nicotinflorin, **C**) narcissin 2

Figure S2. ^{1}H-NMR spectra of flavonoids **A**) Rutin, **B**) nicotinflorin, **C**) narcissin 3

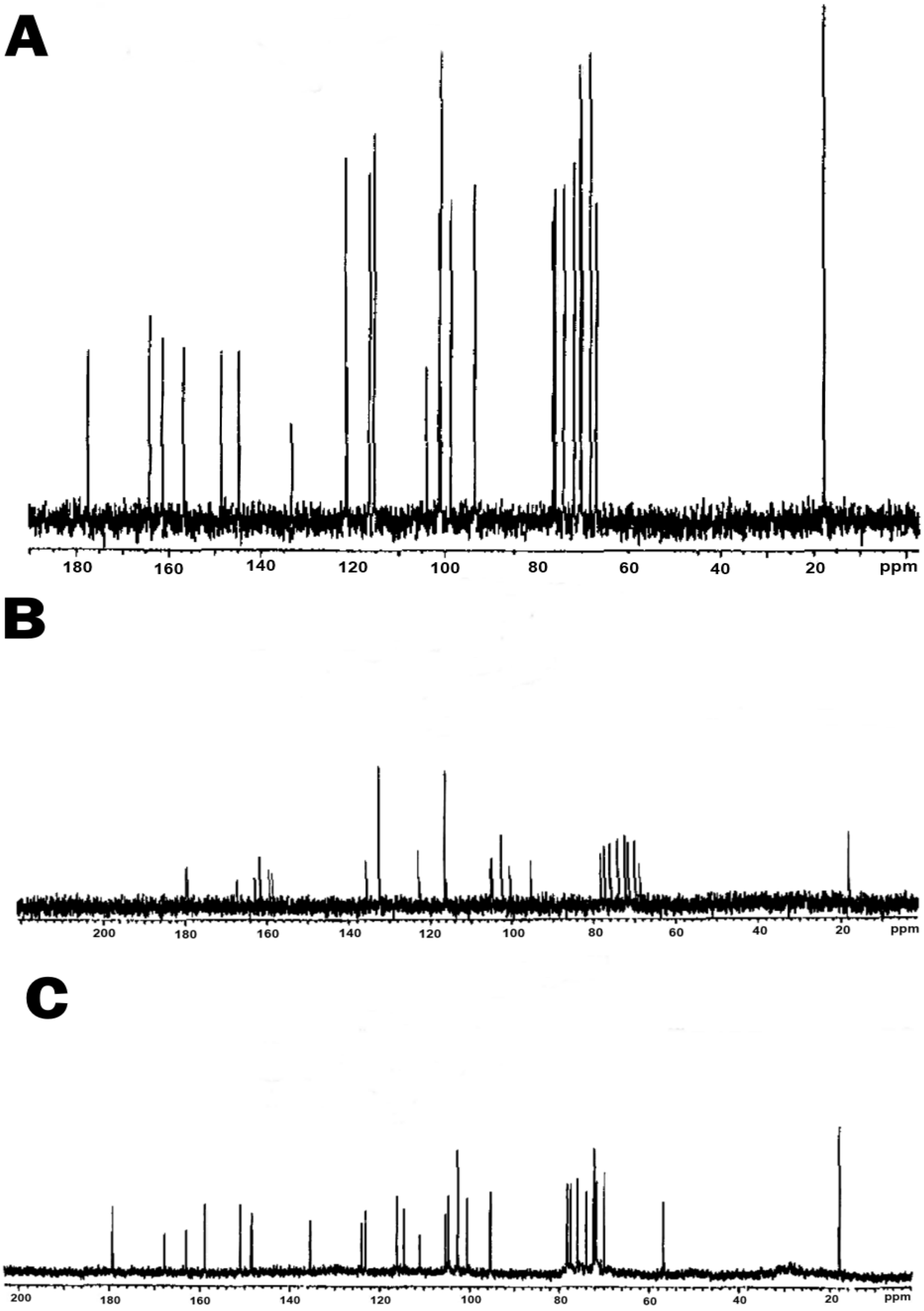

Figure S1. ^{13}C-NMR spectra of flavonoids **A**) Rutin, **B**) Nicotinflorin, **C**) Narcissin

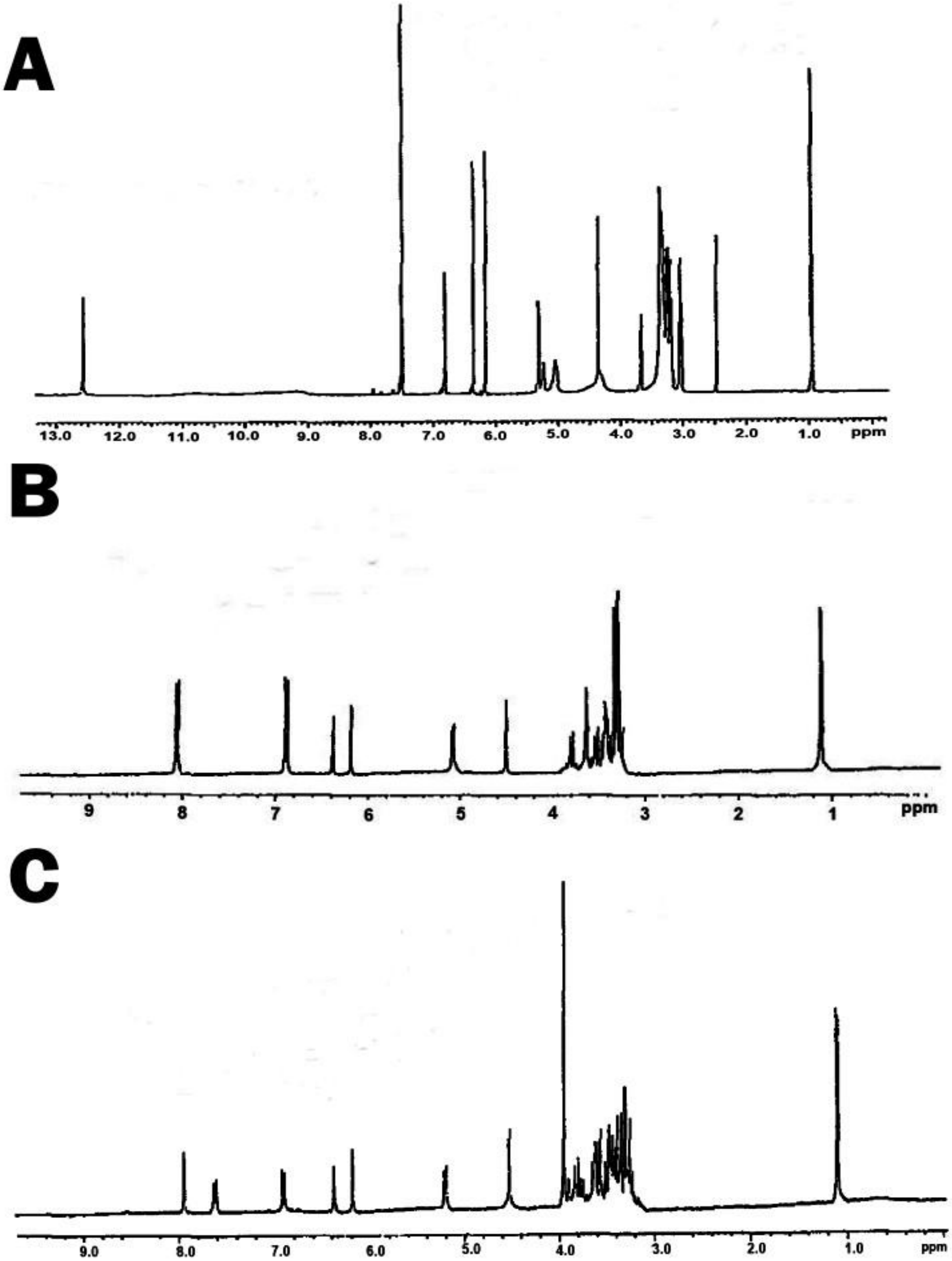

Figure S2. ^{1}H-NMR spectra of flavonoids **A**) Rutin, **B**) Nicotinflorin, **C**) Narcissin.
